# Green and Scalable Manufacturing of Biodegradable Polymer Scaffolds: Solvent-Free Processing, Supercritical CO_2_ and Melt Electrowriting

**DOI:** 10.3390/polym18080974

**Published:** 2026-04-16

**Authors:** Kübra Arancı, Ahmet Akif Kızılkurtlu

**Affiliations:** 1 Department of Bioengineering, Faculty of Engineering, Marmara University, Istanbul 34854, Türkiye; 2 Department of Biomedical Engineering, Faculty of Engineering and Natural Sciences, Istanbul Atlas University, Istanbul 34403, Türkiye

**Keywords:** green synthesis, melt electrowriting (MEW), solvent-free, scalable manufacturing, supercritical CO_2_ (scCO_2_), tissue scaffold

## Abstract

Tissue scaffolds are one of the main components of the tissue engineering triad, playing a vital role in tissue engineering. However, their production procedures heavily rely on solvent-intensive and energy-demanding methods. This raises serious questions about industrial-scale manufacturability, residual solvent toxicity to living health, and sustainability for nature and the environment. Therefore, the main aim of this study is to identify robust scaffolds that provide a suitable microenvironment for resident cells and promote tissue regeneration, while reducing waste through environmentally friendly production methods. In this context, the scalable and ecologically friendly production methods emerge as necessary alternatives as biodegradable polymer scaffolds are used in more therapeutic settings. Clinically applicable and green synthesis-based supercritical carbon dioxide (scCO_2_) technologies, melt electrowriting (MEW), and solvent-free processing techniques are the main topics of this study for a critical analysis of biodegradable polymer scaffold production techniques. Scaffold structure–property correlations, polymer selection and interactions, production procedures, the benefits and drawbacks of existing fabrication technologies, and sustainability issues are discussed in detail. It aims to contribute a novel perspective and approach to literature by presenting and comparing production-oriented approaches as sustainable and green methods. The challenges in the development of biodegradable tissue scaffolds, along with the significance of green manufacturing techniques, are also revealed. The approach is designed to connect processing factors to scaffold features in addition to evaluating current technologies. This review tries to offer a framework for producing biodegradable polymer scaffolds in a sustainable and clinically implementable context.

## 1. Introduction

**Figure 1:**
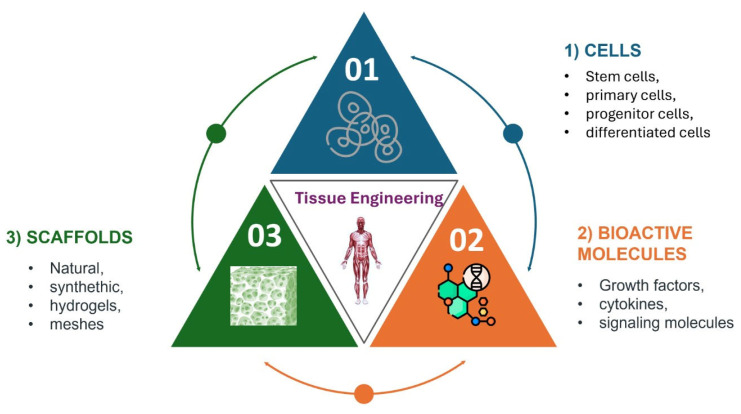
Classic triad of tissue engineering: Cells are responsible for the formation of new tissue through proliferation and differentiation (e.g., stem cells, primary cells, etc.). Bioactive molecules control cellular processes like migration, proliferation, and differentiation. Tissue scaffolds are three-dimensional porous structures, natural, artificial, or hydrogel-based, that serve as a physical template for cell adhesion and mechanical support.

Tissue engineering (TE) is a multi-disciplinary field that matured from fundamental sciences like biology, chemistry, medicine, and engineering disciplines such as bioengineering, chemical engineering, and mechanical engineering. The main objective of TE can be summarized as seeking to restore, replace, or augment damaged tissues [1]. It aims to achieve this crucial goal by integrating cells, bioactive cues, and three-dimensional (3D) scaffolds as biomaterials that provide temporary structural and biochemical guidance (Figure 1). In cases of severe tissue loss, biodegradable polymer scaffolds are considered the first choice because they are able to provide an initial load bearing and space-filling function, while cells can demonstrate progressive adaptation to the system for renewed tissue formation and remodeling. However, obtaining the optimum balance and harmony between scaffold resorption and new tissue generation is a vital point to ensure biocompatibility, and addressing clinically relevant complications, specifically infections, remains a central challenge [2].

While the biomaterials field has delivered remarkable progress in scaffold chemistry, architecture, and biofunctionalization strategy, translation into routine clinical use remains limited by a practical bottleneck: manufacturing. Many established scaffold fabrication routes rely on organic solvents, multi-step post-processing, and low-throughput batch operations [3], all of which complicate reproducibility, quality assurance, and regulatory compliance. On the other hand, sustainability has become a defining constraint of modern healthcare value chains, motivating a shift toward green and scalable manufacturing approaches. It is expected to reduce hazardous substances, minimize process waste, and enable robust production at industrially meaningful throughput without sacrificing scaffold function [4].

In this review, we focus on three complementary manufacturing platforms that are increasingly viewed as enabling technologies for clinically translatable, low-residue, and potentially lower-impact scaffold production. The first is solvent-free melt-based processing, including extrusion-based and related additive manufacturing routes. The second is supercritical CO_2_ (scCO_2_) technologies for foaming and post-processing. Finally, the third is melt electrowriting (MEW) for high-precision micro-architecture. We argue that positioning these methods within a unified green-scale-function idea is timely and necessary to align tissue engineering innovation with the expectations of modern clinical practice.

### 1.1. Global Sustainability Challenges in Tissue Engineering

Sustainability pressures are no longer peripheral to biomedical research and innovation. Instead, they are increasingly central to how healthcare systems, suppliers, and regulators evaluate technologies [5]. The healthcare sector has a substantial climate footprint, estimated to be ~4.4% of global net greenhouse gas emissions, and a large fraction of these emissions is attributable to upstream supply chains rather than on-site hospital operations [6]. This issue is especially crucial for TE, because scaffolds and regenerative implants are often produced via utilizing materials- and energy/labor-intensive processes, followed by complex procurement and sterilization pipelines. Thus, the process is mostly conducted in clean-room settings where single-use consumables are common.

When it comes to a manufacturing perspective for industrial-grade achievement, a key sustainability challenge is the mandatory dependence on organic solvents in polymer synthesis, processing, and formulation. Although TE scaffolds cover a wide range of areas from small-molecule pharmaceuticals, a highly relevant lesson from pharmaceutical manufacturing is that solvent choice often dominates both hazard and waste intensity. Essentially, solvents have been estimated to contribute ~80–90% of the total waste streams, depending on the pharmaceutical manufacturing processes [7]. On the other hand, solvent casting, phase separation, and solution electrospinning can require chlorinated or otherwise hazardous solvents in scaffold fabrication. Moreover, these procedures also suffer from extended drying steps for proper solvent recovery infrastructure and extensive validation of residuals, resulting in increased environmental and operational burden.

In this case, sustainability in TE manufacturing should be considered as a dual obligation. On the one hand, it is reducing environmental and occupational hazards. On the other hand, it is improving the reproducibility and safety of the final implant. It must be noted that utilization of solvents, including methylene chloride and chloroform, has been identified as problematic even in closely biodegradable polymer devices, because of potential environmental and human health concerns [8]. Thus, the solvent type and residual levels are treated as regulated quality issues in relevant biomedical products. This intersection, where green chemistry aligns with patient safety, creates a compelling rationale for solvent-free and low-residue scaffold manufacturing platforms.

### 1.2. Clinical and Regulatory Drivers for Green Manufacturing

Turning lab findings into real-world treatments matters most when building engineered tissues. Because safety counts at every step, materials break down without harming the body. From production to cleanup, each stage needs careful control. While implanted, the structure supports healing yet resists contamination. Function follows form only if chemistry matches biology over time. Even sterilizing methods affect how well it works later. Though built to dissolve, their parts must exit safely after use. Design choices now shape outcomes much farther ahead [9]. If the precautions are not followed in the process, infection-based issues may grow unnoticed. Therefore, the things that are made come into focus when real medical needs arise in terms of not just assembly lines, but actual patient outcomes tied directly to production methods. The structure of the structures emerges based on fabrication choices, where the surface properties vary with the process used, and leftover traces may linger if techniques are not precise. The inclusion of active biological elements inside devices works only under optimum and controlled conditions. As a result, the reproducibility is not optional; rather, it is required to be built into every batch [10].

One reason the industry focuses so much on manufacturing comes from how rules are written, for example, the FDA’s take on ISO 10993-1—biological testing must fit into a broader risk picture, starting with what the device is made of, how it is built, where it goes in the body, and how long it stays there [11]. Over in Europe, laws covering implants demand scrutiny of leftover chemicals, breakdown by-products, and anything that might leach out during use, all tied directly to whether the item is safe. Such demands show that trace materials are not just non-important background issues; they shape the core case for why a degrading scaffold will not harm tissue. Lately, officials have also sharpened their look at how labs identify unknown compounds when checking biocompatibility. As an example, the FDA’s draft document about chemical testing wants to make device evaluations more uniform and trustworthy when checking how safe they are inside the body [12]. On another note, 3D printing now plays a bigger role in creating complicated medical tools shaped exactly for individual patients. With that shift, the agency outlined special steps needed for these printed devices, especially around handling designs carefully and finishing them properly after production.

In addition to residue control and chemical safety, the translational relevance of green scaffold manufacturing must be supported by in vivo evidence addressing host response, vascularization, tissue integration, degradation behavior, and defect-specific functional recovery. Recent preclinical analyses emphasize that scaffold-based constructs may improve matrix organization and defectalso stress that standardized long-term and large-animal studies are required to substantiate clinically relevant claims [13]. In summary, these clinical and regulatory drivers support a clear conclusion where green manufacturing is not only an environmental preference but also a translational enabler. Solvent-free or low-residue-based techniques and procedures can reduce chemical safety issues, reduce variability introduced by solvent removal steps to obtain high reproducibility, and enhance the overall control of the process. Moreover, suitable methods, such as scCO_2_-based sterilization and impregnation, can provide pathways to integrate antimicrobial or therapeutic agents while minimizing thermal or chemical damage to sensitive polymers and biomolecules.

### 1.3. From Laboratory Innovation to Industrial Translation

Regardless of the researchers’ efforts and the advancement of technology, most of the scaffold implementations remain limited to laboratory-scale demonstrations. This is because of the general limitations in throughput, reproducibility, and quality assurance. Thus, the translation gap can be observed especially for porous and micro-architected constructs. In essence, it emerges when the scaffold performance depends on control over pore size distributions, interconnectivity, mechanical integrity, and degradation kinetics [14]. These characteristic features are prone to thermal history, batch-to-batch variability, and post-processing conditions. Hence, the industrial translation requires not only a viable scaffold design but also a manufacturing route for delivering the designs repeatedly, on a determined scale, and under validated controls.

In this context, solvent-free and CO_2_-enabled manufacturing platforms and methods have been increasingly attractive as scalable alternatives to current methods. For instance, supercritical CO_2_ foaming has been described as an innovative green technology in the production of porous polymer scaffolds [15]. Specifically, it is suitable for inducing CO_2_-supported plasticization and controlled nucleation/growth phenomena to generate 3D porosity without conventional toxic solvents. In addition, scCO_2_ has strong potential as a post-processing tool, alongside creating controlled pore formation [16]. The technique is utilized to penetrate polymer matrices efficiently, and scCO_2_-based methods have been explored for terminal sterilization at low temperature. The studies reported the reduced compromise of sensitive biomaterials compared with conventional sterilization routes. This is directly vital for biodegradable scaffolds, which may be vulnerable to heat and radiation-induced chain scission and for which sterilization is unavoidable in clinical use.

**Figure 2:**
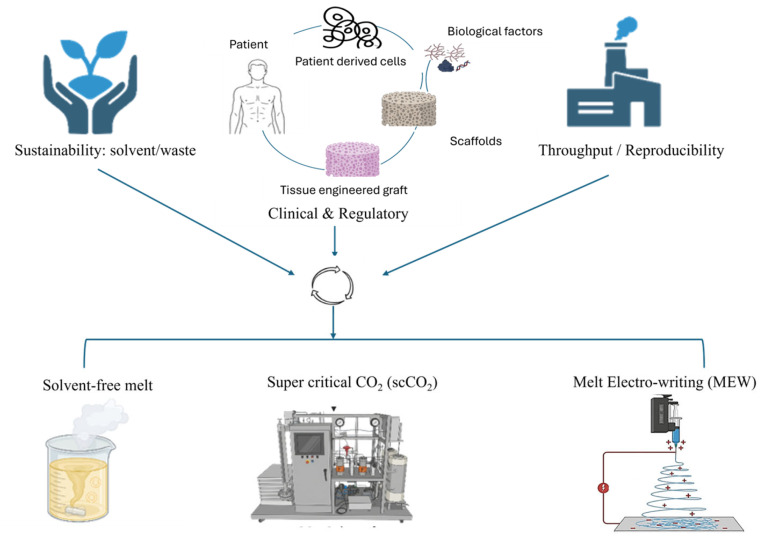
The green-scale-function framework for scaffold manufacturing. Advanced green technologies such as solvent-free melt processing, supercritical CO_2_ (scCO_2_) foaming, and melt electroprinting (MEW) are processes that meet environmental sustainability, clinical/regulatory compliance, and industrial scalability. These processes represent the necessary optimization pathway to bridge the gap between laboratory scale and industrial and clinical application of biodegradable polymer scaffolds.

At the other end of the architectural spectrum, melt electrowriting (MEW) has emerged as a high-precision additive manufacturing technology [17]. It enables highly controlled deposition of micro-scale fibers and customizable scaffold geometries. MEW has been described as an advanced manufacturing route capable of accurate micro-/nano-scale fiber deposition and has gained traction in tissue regeneration [18]. Precisely, it can recapitulate microscale architectural cues that influence cell behavior. However, MEW also highlights a core translation dilemma, where the processes that deliver the finest architectural control can be constrained by production time and throughput, making scale-up, automation, and in-line quality control central research priorities [19]. Therefore, moving from laboratory innovation to industrial translation requires a manufacturing systems perspective, selecting fabrication routes that balance required architecture and function, residue and safety profile, throughput and reproducibility, and compatibility with sterilization and packaging. The most promising pathway for next-generation biodegradable scaffolds may not be a single technique, but rather hybrid workflows, for instance, high-throughput solvent-free melt processing for macro-architecture combined with MEW for micro guidance, and scCO_2_ post-processing for sterilization and/or solvent-free bioactive impregnation. Figure 2 shows the general concept of the green-scale-function framework of this study.

### 1.4. Scope and Structure of This Review

This review focuses on green and scalable manufacturing routes for biodegradable polymer scaffolds intended for tissue engineering. Particularly, we emphasize the solvent-free melt-based processing routes, including extrusion-enabled scaffold fabrication and related solvent-free additive manufacturing approaches. Moreover, we explain the supercritical CO_2_ technologies for scaffold formation (foaming) and post-processing, including sterilization and impregnation concepts. Finally, we discuss melt electrowriting as a solvent-free high-precision microfabrication platform, with attention to its scale-up challenges and opportunities. While we refer to conventional solvent-based methods for comparison where necessary, our primary goal is to identify and synthesize the design rules, process windows, and translation constraints that are specific to low-residue, sustainability-aligned manufacturing. Importantly, we align our discussion with modern synthesis and characterization, clinically motivated constraints such as infection risk, and advanced porous composite scaffolds that may incorporate controlled release of bioactive agents.

The review is organized to move from fundamentals to translation step by step. First, we outline polymer feedstock requirements such as thermal stability, rheology, and CO_2_ sorption that determine feasibility across the targeted manufacturing routes. Afterwards, we provide an in-depth, comparative analysis of solvent-free processing, scCO_2_ technologies, and MEW by highlighting how each platform shapes scaffold architecture, mechanics, degradation behavior, and the feasibility of functionalization. Finally, we address characterization and translational considerations (including chemical safety and process control expectations), reflecting the increasing emphasis of regulatory science on manufacturing processes, residues, and extractables/leachables. The novelty of this review is not merely in describing individual techniques, each of which has been discussed elsewhere, but in providing a unified manufacturing-centric framework that explicitly connects the main parameters. These include sustainability drivers (waste minimization, solvent avoidance, and reduced hazardous inputs), clinical and regulatory drivers (residue control, sterilization compatibility, and chemical characterization expectations), and industrial translation constraints (throughput, reproducibility, process monitoring, and quality-by-design readiness).

By positioning solvent-free processing, scCO_2_, and MEW within a coherent green-scale-function landscape, we aim to offer readers actionable guidance for selecting and combining manufacturing routes. Hopefully, it can deliver advanced biodegradable scaffolds with clinically relevant bifunctionality to accelerate tissue regeneration technologies under real-world constraints.

### 1.5. Definitional Framework: Solvent-Free, Green, and Scalable Are Related but Not Interchangeable

In this review, we will use three terms—solvent-free, green, and scalable—as related but non-synonymous descriptors. In this context, we define solvent-free in a narrow sense as a process that produces a scaffold without using organic solvents to form a scaffold or assemble it. This definition is particularly relevant for biodegradable and medicated scaffolds, as it directly addresses some of the risks associated with residual-solvent toxicities, the need to purify the scaffold from solvents, and the inactivation or loss of bioactive agents during the extraction or solvent-removal steps. While being solvent-free may encompass several process performance metrics beyond solvent use alone, including feedstock source, energy requirements, equipment requirements, package and sterilization requirements, and total lifecycle emissions, it is not enough to adequately define the overall sustainability of a process.

By contrast, green is intended to encompass a broader environmental sustainability context, as defined in green chemistry and engineering frameworks. Rather than simply indicating a lack of solvent, green encompasses many aspects of a process’s entire lifecycle, including the reduction in toxic chemicals, waste, unnecessary auxiliary chemicals, and, where practical, energy and natural resource consumption. In reference to scaffold manufacture, we will consider a process to be green if it provides the opportunity for substantial reductions in all the environmental and safety-related burdens associated with it, rather than merely avoiding the use of solvents. For example, a process can be considered green if it uses renewable energy sources, produces fewer hazardous compounds, utilizes recycled process fluids, decreases the overall process intensity, or affords more favorable lifecycle analyses. Thus, while solvent-free methods may provide a pathway to greener scaffold manufacturing, they do not represent a complete definition of green manufacturing.

Lastly, scalable is used as a descriptor for both manufacturing methods and the degree to which a process can be successfully implemented in an industrial context. In this review, a process is scalable if it can be scaled up while retaining the essential quality attributes of the scaffold, consistent production of scaffolds, effective control of the process, appropriate regulatory compliance, and economically viable throughput. In this sense, scaling a process refers to more than increasing the size of the scaffold being manufactured; it also indicates the extent to which a process can be transitioned from laboratory- to commercial-scale manufacturing settings in a controlled manner. Thus, a process can be scalable without being environmentally sound, as a greener process may be more difficult to scale due to its long production cycle times, specialized machinery, or limited robustness. To distinguish between these concepts, the remainder of this review will utilize the term solvent-free for chemistry/processes, green for environmental sustainability, and scalable as descriptive of industrial manufacturability.

## 2. Biodegradable Polymers Suitable for Green Scaffold Manufacturing

Biodegradable polymers are materials that break down through living microorganisms or environmental factors, producing environmentally harmless or minimally harmful products such as water, carbon dioxide, or biomass. They are classified into two main categories based on their origin: synthetic and natural [20].

### 2.1. Synthetic Biodegradable Polymers

Synthetic biodegradable polymers—representing the broadest group of biologically degradable polymers—are polymers that can be designed and synthesized with more precise control under physical and chemical conditions [21]. Their biocompatibility properties, adjustable characteristics compared to natural polymers, and the fact that they produce non-toxic degradation products when they degrade over time through hydrolysis, enzymatic degradation, or oxidative mechanisms have led to growing interest in synthetic polymers in recent years. Synthetic biodegradable polymers frequently used in green scaffold manufacturing technology include Polyvinyl alcohol (PVA), Polycaprolactone (PCL), Polylactic acid (PLA), Poly (lactic-co-glycolic acid) (PLGA), Polyurethane (PU), Polyethylene oxide (PEO), and polyethylene glycol (PEG) [20].

Importantly, the suitability of a synthetic polymer for scaffold manufacturing should not be generalized only by polymer family name, because grade-dependent descriptors often govern both processability and degradation. For this reason, parameters such as the degree of hydrolysis and molecular weight for PVA, molecular weight and crystallinity for PCL, stereochemistry and crystallinity for PLA, and lactic:glycolic ratio, molecular weight, and end-group chemistry for PLGA should be considered when discussing material selection.

Poly (vinyl alcohol) (PVA) is a polymer that is categorized as hydrophilic. This means that when it encounters water, it attracts and absorbs moisture and can completely dissolve in solution through hydrolysis of poly (vinyl acetate). The physicochemical properties of PVA will differ considerably depending on the molecular weight of the polymer and the degree of hydrolysis. As such, higher-molecular-weight PVA has greater tensile strength, improved resistance to water and solvents, and is characterized by greater crystallinity than lower-molecular-weight PVA. Conversely, PVA that has been partially hydrolyzed contains residual acetate groups and, therefore, retains some solubility in water and enhances processability. The degree of hydrolysis of PVA is critical to the use of PVA as a scaffold for aqueous scaffold fabrications, swelling control, and the design of physical or chemical cross-linking PVA-based structures [22,23].

Poly(caprolactone) (PCL) is an aliphatic polyester that is semi-crystalline and has low melting and glass transition temperatures. This feature makes it an attractive scaffold material to fabricate using melting. The biodegradation of PCL should not be viewed as a constant value; however, it depends on many factors, including but not limited to molecular weight, crystallinity, end-functional groups, geometrical profiles, and the environment that the construct is being subjected to during biodegradation. In general, the biodegradability of higher-molecular-weight PCL and more crystalline PCL is slower because water penetration and chain cleavage are hindered. Conversely, lower-molecular-weight PCL and structures with higher surface areas are typically much quicker to degrade. Molecular weight-dependent degradation of PCL can contribute to PCL’s suitability for providing long-term structural support, but it will often necessitate the blending of PCL to give the necessary properties or a modification of PCL’s chemical composition to achieve more rapid scaffold resorption [24,25,26].

Polylactic acid (PLA) is a thermoplastic polyester whose performance is affected by both its molecular weight and its stereochemical structure and degree of crystallinity. The molecular weight, stereochemical structure, and degree of crystallinity influence PLA’s stiffness, brittleness, thermal properties, hydrolysis, and processability. For example, because of this combination of factors, the behavior of amorphous, semicrystalline, and crystalline grades of PLA will vary during scaffold fabrication and degradation. Compared to PLA, the lactic: glycolic acid ratio, molecular weight, and terminal functional groups of polylactic-co-glycolic acid (PLGA) create an expanded design space for degradation. The more lactide in the formulation, the more hydrophobic it is, and subsequently, the longer the degradation time. Conversely, the greater the amount of glycolide that is present in the formulation, the more quickly that formulation will take up moisture and degrade. The 50:50 ratio of PLA to PLGA has consistently been reported to have the shortest degradation time. Higher-molecular-weight PLGA grades, like PCL, typically have strength properties that exceed those of lower-molecular-weight PLGA, whereas PLGA with acid-terminated functional groups degrades faster than PLGA with ester-terminated functional groups. Therefore, the choice of polymer for scaffold design should be considered in terms of the relationships between the polymer composition, polymer structure, and polymer processing rather than polymer classifications or specific polymer names [27,28,29].

PEO/PEG are polymers that share the same carbon chain backbone (ethylene oxide chain) but differ in synthesis method and molecular weight. PEG refers to polymers with a molecular weight below 20,000 g/mol, while PEO refers to polymers with a molecular weight above 20,000 g/mol [30]. PEG is a water-soluble, non-ionic polymer that is biodegradable by the addition of functional groups to its structure. Due to its biocompatibility and non-immunogenic properties, it is widely used in tissue engineering applications. For example, its use is approved by the FDA to limit ischemia/reperfusion injury before organ transplantation. In another example, the process of coating the surfaces of biomaterials with PEG is called Pegylation [20]. With the help of this process, nanostructured scaffolds or cell-carrying nanoparticles are not perceived as foreign substances by the immune system, the immune response is reduced, and rapid degradation is prevented, thus increasing therapeutic efficacy. PEG has been shown to play significant roles in spinal cord injuries by inhibiting the inflammatory response, suppressing microenvironmental changes, crossing the blood–brain barrier, and facilitating cell therapy and tissue regeneration [31,32].

#### Beyond Homopolymers: Block, Graft, and Segmented Copolymer Architectures

While homopolymeric scaffolding materials such as PCL, PLA, and PVA remain prevalent, the area of fabrication is not restricted to solely homopolymeric systems. Whenever one homopolymer does not satisfy all requirements associated with the printability, wettability, elasticity, degradation profile, and biological performance of scaffold production, copolymerization architectural design can be employed. PLGA is an example of a material that is copolymeric in nature, and the combination of lactide and caprolactone in PLCL permits a tunable elastic profile, tunable degradation behavior, and compatibility with various processing routes, including 3D printing and scCO_2_-based fabrication. In general, amphiphilic block copolymers, such as the PEG–polyester systems, have been discussed frequently, as they can be used to tailor the swelling of scaffolds, degradation behavior, handling, protein adsorption, and cell interactions with the scaffold [33,34,35].

The use of these architecturally defined copolymers is becoming increasingly important in both melt-based additive manufacturing and high-precision additive manufacturing as they increase the variety of biomaterials that can be processed, as well as providing fine-tuned control of the functions of scaffolding. As evidence of this, recent investigations indicate that PEOT/PBT multiblock copolymers can be melt-electrowritten into stable scaffolds with tunable physicochemical and mechanical properties, while maintaining the ability to support cell adhesion and proliferation. Amphiphilic segmented copolymers possessing both hydrophilic and hydrophobic blocks can be processed by melt electrowriting. There has been a systematic investigation of the morphology, mechanical properties, cytocompatibility, and cell adhesion behavior of scaffolds produced from these segmented copolymers. The research presented in these investigations supports the concept that block, multiblock, and segmented copolymers should not simply be viewed as exceptions to scaffolding materials formed from homopolymers. Instead, they should be studied as both intentional and architectural design platforms for 3D-printed scaffolds [36,37,38].

Graft architectural design also has significance in scaffold preparation, specifically when compatibility at the interfacial level, ease of processing, or surface biofunctionality is to be improved. Specifically, by employing PLA/CS-g-PLA scaffolds, it has been demonstrated that improved phase compatibility exists, hydrolytic stability is improved, and cell adhesion and growth of the MG-63 osteoblast and fibroblast cells occur better than with scaffolds composed of non-compatibilized PLA/CS polymers only. In a recent study, the grafting of L-lactide onto chitosan produced a thermoplastic chitosan–PLA copolymer, characterized as melt-processible, having adequate mechanical properties, being non-toxic, and possessing improved fibroblast adhesion, indicating that, through graft copolymerization, difficult-to-process polymers can be effectively utilized as materials for producing scaffolds. In summary, scaffold preparation should be defined as being comprised of both homopolymers and architecture-engineered copolymers, with the design of macromolecules serving as an independent variable that works in conjunction with the polymer identity, molecular weight, crystallinity, and processing route [39].

### 2.2. Natural Biodegradable Polymers

Macromolecular structures that can be obtained from both renewable and biological sources, such as animal, plant, marine, and microbial sources, and that can be broken down over time by microorganisms are called natural biodegradable polymers [21,40]. Generally, they are classified under three main titles: polypeptides (such as collagen, gelatin, and silk fibroin), polysaccharides (cellulose, chitosan, alginate, carrageenan, starch, and hyaluronic acid), and polyesters. Their most important characteristics are excellent biocompatibility, availability from biological sources that can be broken down by enzymes, and the fact that they release non-toxic degradation products into the environment. Furthermore, due to the high similarity of their specific molecular structures to the body’s natural extracellular matrix (ECM) components, they play a significant role in accelerating wound healing

Cellulose is the most abundant agro-biopolymer in nature. It is found in lignocellulosic plant cell walls and contains numerous -OH groups in its structure. These functional groups interact with chemical reactants, forming strong hydrogen bonds and playing a significant role in the formation of new cellulose derivatives, the acquisition of crystalline structures, and the improvement of mechanical properties. Cellulose acetate and cellulose esters are the most common types of cellulose derivatives. They generally have diverse application areas, including wood products, textiles, paper, food, pharmaceuticals, and cosmetics [40,41,42].

Chitin is the second most abundant polysaccharide in nature after cellulose and is also known as an amino polysaccharide. It is a polysaccharide found in the cell walls of crustaceans such as shrimp, insects, crabs, and lobsters, as well as fungi, providing structural support [40]. Although structurally like cellulose, chitin has an acetamide group at the C_2_ position instead of a hydroxyl group in its glucose unit. This structure contributes to high crystallinity, the formation of strong hydrogen bonds, and the formation of antiparallel nano-sized chitin nanofiber structures [43]. However, the strong intra- and intermolecular hydrogen bonds in both cellulose and chitin make them difficult to dissolve in common solvents, creating obstacles in their processing and conversion into biological products. Therefore, their application areas are limited. However, like cellulose, chitin also has a derivative called chitosan, which is formed by deacetylation. Chitosan is produced by removing the acetyl groups from chitin and forming amine groups, and chitosan has higher solubility than chitin under acidic conditions [44,45]. Chitin and chitosan have a wide range of applications in biomedical fields due to their advantages, such as biocompatibility, biodegradability, non-toxicity, and non-immunogenicity. For example, the positive charges of chitosan impart antibacterial properties by disrupting the cell walls of bacteria, causing their death, while these charges also enhance drug interaction by forming hydrogen and ionic bonds with some drugs, facilitating drug delivery to the target area. In this context, chitosan shows high suitability for use in nanoparticulate drug delivery systems. Furthermore, chitosan exhibits rapid hemostatic properties by binding negatively charged red blood cells and, due to its clotting-promoting properties, is an important component in the development of wound dressings and tissue scaffolds [46].

Alginate is a biopolymer primarily obtained from the cell walls of brown seaweed and is quite abundant in the marine environment. Alginate is an anionic polymer with low toxicity, high biocompatibility, and biodegradability, exhibiting rapid gelling ability. However, when alginate encounters divalent cations such as Ca^2+^, it hardens very quickly, creating a platform for hydrogel structures. In recent years, its widespread application in fields such as tissue engineering, drug delivery, and wound-dressing material development has accelerated due to the high similarity of alginate-based hydrogels, developed with different cross-linking techniques, to ECMs [47].

Carrageenan is a polysaccharide found in red seaweed species, containing linear and highly sulfated galactans in its structure. Its most important feature is its high similarity to glycosaminoglycan (GAG) structures found in the ECM of connective tissues, improving cell adhesion and proliferation and offering unique properties for tissue scaffold designs. Studies on carrageenan’s immunomodulatory, antibacterial, antiviral, antioxidant, and anticoagulant properties have led to its recommendation for use in controlled drug delivery systems, cell encapsulation, and wound-dressing materials, as well as in tissue engineering applications, particularly in cartilage and bone tissue engineering [48,49].

Collagen is the most abundant protein in humans and animals, forming part of the extracellular matrix (ECM) (ECM) and many tissues. Its triple helix structure and peptide chains provide a natural microenvironment for cell adhesion, proliferation, and differentiation. Its highly biocompatible, biodegradable, porous, and weak antigenic structures make it crucial in the development of cell-friendly tissue scaffolds. Collagen acts as a matrix that mimics biological signals and directs tissue regeneration, particularly in bone, cartilage, skin, and nerve tissue engineering research. Furthermore, the risk of post-implant inflammation can be reduced by regulating the natural rate of collagen degradation to be compatible with the tissue regeneration process [50].

Gelatin—a natural and biodegradable biopolymer—is produced by hydrolyzing collagen’s triple-helix structure to its single-stranded form. Because of its excellent biocompatibility, gel-forming ability, and hydrophilic properties, it is frequently employed in drug delivery systems, injectable hydrogels, and tissue engineering applications [51]. Gelatin has significant functions in cell adhesion, proliferation, and differentiation because of the RGD (Arg-Gly-Asp)-like biological patterns in its structure that support cell adhesion. Its mechanical strength is limited by its hydrophilic nature, although it can be improved by employing various chemical or enzymatic cross-linking techniques. By allowing the controlled release of substances like medications and growth hormones, gelatin speeds up the regeneration process. It is often employed in tissue engineering applications for skin, bone, cartilage, and nerves because of these qualities [52].

### 2.3. Polymer Blends and Composite Systems

**Figure 3:**
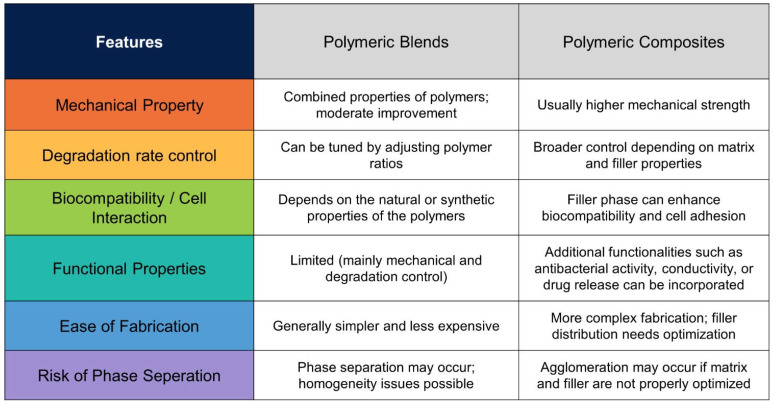
Comparison of blending and composite biodegradable polymer structures.

Biodegradable polymers used alone have limited ability to replicate the mechanical strength, biocompatibility, biodegradation rate, and natural ECM architecture required for green scaffold production. To overcome these disadvantages, the need for polymer blending or the addition of reinforcing materials has emerged as a critical approach. Polymeric blends are structures formed by combining two or more polymers without any chemical bond between them to achieve desired properties or characteristics. Composite structures are multi-phase systems in which a reinforcing phase (fiber, particle, etc.) with different properties than the polymer is dispersed within a polymeric matrix. The aim of both blends and composite structures is to improve the physical, chemical, and mechanical properties of existing biodegradable polymers [53]. Blending and compositing structures are cheaper, require less energy, and align with sustainable production policies compared to new polymer development efforts [54,55]. A comparison of the advantages of both blending and composite structures is summarized in Figure 3. In recent years, there has been increased interest in adapting blend and composite structures to performance and market demands [42]. For example, blending biopolymers with slow degradation rates with highly degradable polymers can lead to the development of materials with a specific level of degradation for targeted applications. A representative example is PLA polymer, which has high mechanical strength but limited applications due to its low hydrophilicity, high brittleness, lack of elasticity, and crystalline structure. However, in structures formed by blending PLA polymer with flexible polymers such as polyhydroxyalkanoates (PHA) and polybutylene adipate terephthalate (PBAT), flexibility is increased while maintaining mechanical strength. Alternatively, the degradation kinetics and brittleness of PLA can be improved by using polymers with a lower glass transition temperature, such as PCL, while the degradation kinetics can be controlled by using hydrophilic polymers such as PEG [56,57].

The development of composite materials is a strategy used not only to regulate mechanical properties and biodegradation rate, but also to overcome the disadvantages of synthetic polymers, which have insufficient biological activity, low cell binding capacity, and limited surface cell recognition properties. Although synthetic polymers have advantages in terms of processability and mechanical properties, they cannot meet all the criteria for tissue regeneration on their own. Therefore, composite scaffolds derived from natural polymers and synthetic biodegradable polymers have the potential to meet many clinically important requirements, such as the size and type of wound, the patient’s needs, and the manufacturing methods used [58]. For example, in a periodontal regeneration study, adding gelatin and chitosan to PCL polymer increased hemostatic properties while creating porous structures, accelerating wound healing by increasing blood absorption, nutrients, and oxygen diffusion [59]. In another study, tissue scaffolds developed by adding cellulose nanocrystals to PLA polymer, used in bone tissue engineering applications, exhibited very good cell adhesion and mineralization, and bone regeneration increased in the scaffold-treated group [60]. In a study on nasal cartilage and subchondral bone reconstruction, adding gelatin and osteogenic drugs to the surface of 3D-printed Poly (L-lactic acid) (PLLA) scaffolds increased mineralization and biological activity [61].

Numerous examples of composites derived from natural–natural, synthetic–natural, and synthetic–synthetic polymers have been reported in the literature. However, when selecting materials for tissue engineering scaffolds, the individual advantages and limitations of each material should be carefully considered, and the materials should be tailored to meet specific clinical requirements [58,62].

### 2.4. Structure–Processing Properties Relationships

Ideal tissue scaffolds should possess appropriately scaled interconnected pores, controlled biodegradability or absorbability, and a surface capable of facilitating cellular adhesion, growth, and differentiation, exhibit sufficient mechanical properties for implantation, and not cause allergic reactions. They should be producible in desired shapes and sizes. When examining the structural characteristics of successful tissue scaffolds, micro-scale structures are critical for creating a suitable microenvironment for cell survival and function. In contrast, macro-scale tissue architecture creates structural integrity that allows for the coordination of multicellular processes, ensures adequate nutrient and oxygen transport, and provides the necessary mechanical properties [63,64]. In particular, parameters such as pore size, pore shape, and porosity, which are microstructures of tissue scaffolds, are among the most important parameters contributing to cell growth, migration, differentiation, and dynamic stability in tissue scaffolds. To achieve a balance between tissue scaffolds and their functions, appropriate tissue scaffold production methods must be selected. There are many production methods for producing desired tissue scaffolds with different properties and structures. However, the processing methods of tissue scaffolds should be selected considering parameters such as porous structure formation, improved mechanical strength, cell adhesion, and biodegradability in the materials.

**Figure 4:**
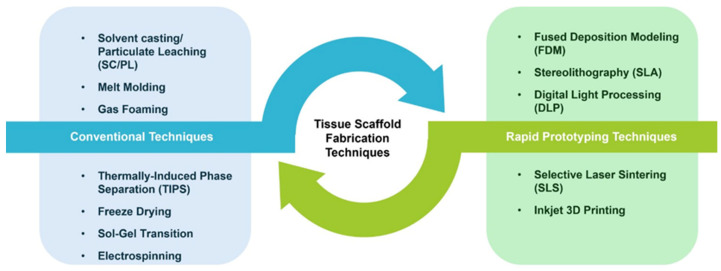
Tissue scaffold fabrication techniques.

Tissue scaffold production is examined under two main titles: conventional and additive manufacturing (Figure 4). Solvent casting and particle leaching, phase separation, gas foaming, freeze-drying (lyophilization), and electrospinning are the most frequently used conventional methods [48]. Conventional methods aim primarily at creating porous structures and shaping polymers. However, producing large-volume scaffolds with complex geometries using these methods is quite difficult. Pore size and connection controls are irregular and limited. Mechanically, generally unstable scaffolds are formed. To overcome these limitations, more modern 3D printing methods have been developed, resulting in more stable, functionally customized scaffold designs. These methods, developed using computer-aided design (CAD) programs, are called rapid prototyping. This method is also an additive manufacturing (AM) approach, as raw materials, whether solid powder or liquid, are added layer by layer and solidified [63].

In recent years, various 3D printing processes have been developed that allow control over many properties of scaffold composition. These approaches, along with the increasing variety of CAD software (e.g., SolidWorks, AutoCAD, and similar platforms; versions vary depending on application) and biomaterials, have made it possible to design and produce patient-specific scaffolds with complex 3D structures. The most well-known 3D printing methods are digital light processing (DLP), fused deposition modeling (FDM), inkjet, selective laser sintering (SLS), and stereolithography (SLA) [4]. Both SLA and DLP production methods share the common characteristic of being light-based. SLA, one of the oldest known methods, uses a UV laser to cure photopolymer resin layer by layer. In contrast, DLP uses light projection (2D light projection) to cure the photopolymer resin layer by layer. This projection allows for the creation of the entire 2D segment of the object in a single pass, making it faster. In SLA, however, layers are cured individually, making the process slower, but the resolution is higher than DLP [4,65,66].

SLS is a printing method where powdered polymer, metal, or ceramic particles are sintered layer by layer with the help of a laser to create a 3D solid model. They are generally preferred for bone and hard tissue scaffolds because the materials produced have high mechanical strength. They can be used in the production of complex structures. However, their resolutions are considerably lower compared to DLP/SLA. Equipment costs are also very high [67,68].

The most used manufacturing method in the production of tissue scaffolds is FDM, an assembly-based manufacturing method. Thermoplastic polymer filament is melted through a heated nozzle, and a three-dimensional structure is created by depositing it layer by layer according to CAD. It is less expensive and easier to use compared to other methods. Its most important advantage is that it allows the production of structures with large pores. However, because high temperatures are used to melt the polymer, it is not suitable for the direct printing of cells or biological molecules [69].

As previously mentioned, properties such as pore size, interpore connection, and pore volume must be suitable for the target tissue. Suitable polymers, the best morphological structures, and the most appropriate manufacturing methods must be selected to produce tissue scaffolds that meet the requirements of the target tissue, considering the merits and drawbacks of each manufacturing method [70].

### 2.5. Processing Windows for Printability, Biocompatibility, and Cellular Responses

The optimized production conditions across all of the manufacturing processes presented (in this study) should be used to define the “process window”—also referred to as the “process parameter”—for each manufacturing process and define a range of working conditions (or processes) over which each manufacturing process can produce the desired construction. Printability in the additive manufacturing process is not just about the ability to extrude or deposit a material; printability also means that when printed, the printed object holds its shape, maintains its integrity after printing, and remains structurally stable. The body of printability literature connects success in printing to rheological characteristics, recovery after deposition, manufactured object reproducibility, and continued appropriateness for a biological function. A tissue scaffold’s printability should be determined together with the design goals, degradation profile, and sterilization process, rather than solely defining a physical geometry [71]. In the case of producing scaffolds from solvent-free melt processing and from the extrusion of a polymeric material into a scaffold, the capability of producing the printed object is to continue to operate above the melting/softening temperature of the polymer; to operate below the temperature range that creates excessive flow and creates an incomplete layer bonding; and to be able to maintain the plasticity of the printed object. The tradeoff between the two extremes has been documented in a recent study on polycaprolactone/bioactive glass scaffolds; the authors discovered that using an extrusion temperature of 90 °C resulted in extended printing times with failure to achieve the architecturally designed geometry, while using an extrusion temperature of 130 °C resulted in significant geometric deviations from the intended geometry. Only at 110 °C, and on average, did the authors get the closest pore sizes to what was designed and produce scaffolds of the same consistent quality [72]. In summary, printability in the process of manufacturing scaffolds from the melt is not only about the temperature of the nozzle. Nozzle temperature, how the object is built, the use of support materials, and heat control throughout the process all contribute to the determination of the printed scaffold, and thus, printability is more of a multi-variable process versus a single material/temperature parameter. With respect to the process window of MEW, melt electrowriting is much tighter than the electrospinning process. The process window for MEW is limited by the maintenance of a stable Taylor cone and the ability to write directly above the critical translation speeds. The modeling and review literature reveal that the following variables are the primary variables influencing jet stability: fiber diameter (size), accuracy of deposition, and pore regularity: melt temperature (of the material), feed rate (pressure), applied voltage, nozzle to collector distance, collector speed, and environmental conditions [73]. Small deviations in these parameters can significantly impact on the ability to write in a straight line and therefore lead to jet lag and/or uneven fiber placement or the instability of the jet. As such, optimal printability of MEW is better described through coordinated parameter combinations that balance electrohydrodynamic stability and positional accuracy rather than optimizing individual parameters in isolation.

While printability is a concept unique to additive manufacturing, by analogy, the equivalent means to evaluate the printability of a non-printed route, i.e., supercritical CO_2_ processing, is morphology fidelity and controlled (uniform) pore generation. The combination of saturation pressure, temperature, soaking time, and depressurization rate information relates to pore nucleation and pore interconnectivity and the potential for dense skins to form on the exterior of the scaffold. The results of recent studies have shown that hydrothermal pre-foaming treatment increased scaffolds’ porosity and interconnectivity; however, the results of studies also show that post-foaming treatment may lead to pore collapse and structural integrity loss [74]. Therefore, optimization of supercritical CO_2_ processing may, in turn, represent a time-sensitive process window in which the mechanical and morphological properties of the material, the mechanical function of the device, and biological functions perform together as one, thereby providing the optimal means of achieving the result.

A variety of combinations of conditions influence optimal biocompatibility and cellular response; however, these conditions are also dependent on processing and specific to a particular tissue type. The literature currently available regarding scaffolds indicates that there are a variety of factors that affect nutrient transport, the adhesion and migration of cells within scaffolds, as well as their mechanical behavior: the pore size, pore geometry/shape, interconnectivity, and porosity, and there is no well-defined and universally optimal pore size for all cell types and tissues [75]. Macropore size greater than 300 microns has been shown to provide improved adhesion, proliferation, and vascularization for osteoblasts; however, gradient pore structures have shown improved performance relative to uniform pore scaffolds for many applications. In addition to the physical architecture of the scaffold, the thermal processing history provides additional critical information regarding crystallization behavior, crystal size distribution, surface roughness, and surface stiffness; these factors will greatly influence the attachment, proliferation, and differentiation of cells. In semi-crystalline thermoplastics, the above features are directly controllable by the manufacturing conditions; print settings can potentially become cell-instructive variables. In addition to processing, the consideration of surface chemistry and degradation behavior while defining the biologically favorable manufacturing conditions is critical. For instance, PCL is still a popular material for manufacturing because of its ease of processing and mechanical stability; however, due to its hydrophobicity, cellular attachment and proliferation are negatively impacted unless surface modification or bioactive surface treatments occur. Review articles published in recent years demonstrate that there is a correlation between chemically treated scaffolds and improvements in surface roughness, wettability, and cell response; however, too much treatment could negatively affect the mechanical properties or filament integrity of the printed object. In general, both the literature regarding scaffold designs as well as the literature regarding bone scaffolds clearly indicate that degradation of scaffolds should occur synchronously with the formation of new tissue to allow the scaffold to provide transient structural support without interfering with the natural remineralization process or releasing unacceptable degradation products [72]. In addition, proper sterilization conditions are extremely important; biological performance is influenced not only by cytocompatibility chemistry but also by the maintenance of architectural integrity, surface functionality, and stability of the raw material after completion of the manufacturing cycle. Thus, the optimal condition for the manufacture of scaffolds must be determined by balancing manufacturability, biocompatibility, and cell instructive performance; therefore, the optimal condition for manufacturing scaffolds will not necessarily be at the highest print fidelity.

## 3. Green and Scalable Manufacturing Methods for Tissue Scaffold Production

Traditional and rapid prototyping production methods used in tissue scaffold manufacturing have many advantages and disadvantages. For example, the organic solvents and chemicals used in traditional production methods are toxic. They can also cause inflammation or death in cells when they come into contact with them. Similarly, some photoinitiators and solvents used in rapid prototyping systems can be toxic. Both production methods lead to environmental pollution and chemical waste problems with industrial-scale production. In traditional methods, production processes are slow, and mass production is difficult. Even with repeated production at the same parameters, it is quite difficult to obtain results of the same quality each time. In rapid prototyping systems, the equipment is quite expensive, and some systems require high energy. Due to these limitations, in recent years, interest in green synthesis strategies, which offer environmentally friendly, low-toxicity, and industrially applicable approaches in tissue scaffold manufacturing, has increased [76,77,78].

**Figure 5:**
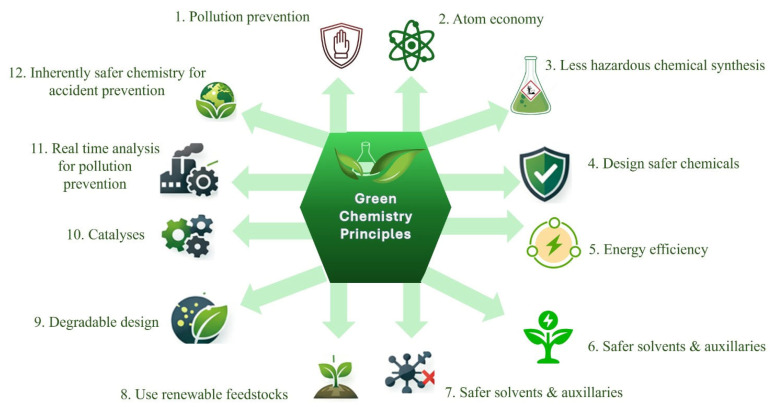
Green chemistry fundamentals.

Greener nanomaterials refer to nanomaterials that are designed, synthesized, and used to reduce their environmental impact, decrease toxicity, and promote sustainability. These materials are typically produced using environmentally friendly methods, such as green chemistry principles. These are intended to contribute to more sustainable technological applications while minimizing harm to both human health and the ecosystem [76,79]. Founded in the 2000s by Anastas and Warner, green chemistry has 12 fundamental principles [78,80]. These principles are summarized in Figure 5.

**Figure 6:**
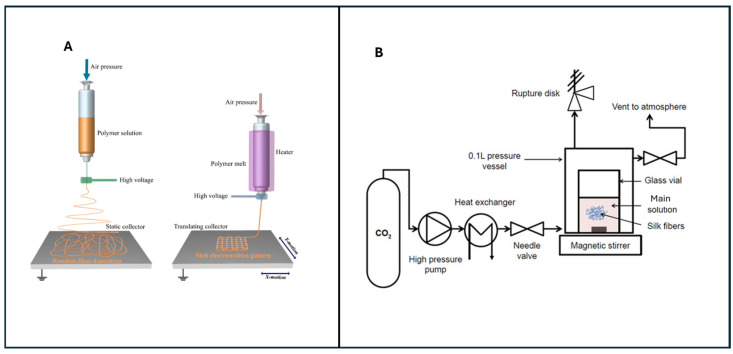
(**A**)The schematic difference of melt electrowriting (right side) over electrospinning (left side) [84]. (**B**) Schematic diagram of the supercritical CO_2_ setup [85].

Green synthesis methods reduce or eliminate solvent use. Eco-friendly materials with increased biocompatibility are produced. This contributes to the production of more cost-effective materials, consumes less energy, and is more suitable for industrial production compared to traditional methods, all within the framework of sustainability principles [81]. The most commonly known green synthesis methods for tissue scaffold production are solvent-free [82], supercritical CO_2_ [83], and melt electrowriting methods [17]. The three different ways to make products, mentioned above, use different types of substances and have different effects on the environment, and can be made in larger quantities than any of them by the way feedstocks are chosen, process conditions used, energy demands, post-processing storage, and quality-control methods employed. Figure 6 shows the supercritical CO_2_ and melt electrowriting method concepts.

## 4. Solvent-Free Manufacturing Routes for Biodegradable Polymer Scaffolds

Solvent-free scaffold manufacturing encompasses processing routes in which the three-dimensional (3D) construct is formed without the use of organic solvents during scaffold assembly, thereby minimizing risks associated with residual solvent toxicity, solvent recovery infrastructure, and process-related variability [70]. In the context of biodegradable polymer scaffolds, this family of approaches includes melt molding (compression, injection, and extrusion molding), material-extrusion additive manufacturing (commonly referred to as fused deposition modeling/fused filament fabrication), sintering-based consolidation (thermal, CO_2_-assisted, or laser-driven), and gas/CO_2_ foaming strategies [86]. Notably, these solvent-free techniques have been emphasized as promising for clinically oriented scaffolds because they can reduce purification steps and, for drug- or factor-loaded constructs, can mitigate the “washing-out” losses often associated with solvent-based post-processing.

Within this section, we focus on solvent-free processing routes that are most directly compatible with industrial translation: melt extrusion/melt molding and solid-state or low-temperature solvent-free methods (Section 4.2). Supercritical CO_2_-enabled processing is discussed in depth in a dedicated section of this review (Section 6), but here we briefly introduce CO_2_-assisted solid-state routes where they serve as genuinely low-temperature, solvent-free consolidation methods.

### 4.1. Melt Extrusion and Melt Molding Approaches

Melt-based processing routes rely on thermoplastic flow above the polymer’s glass transition temperature (Tg) for amorphous polymers or above the melting temperature (Tm) for semi-crystalline polymers [87]. Classic melt molding includes compression molding, injection molding, and extrusion molding, while melt-based additive manufacturing typically employs material extrusion through a heated nozzle to deposit filaments layer-by-layer. These approaches are widely considered among the most scalable and reproducible manufacturing options for polymeric medical devices and have been highlighted as cost-effective and industrially mature for polymer processing.

#### 4.1.1. Melt Molding: Compression Molding, Injection Molding, and Extrusion Molding

In melt molding, polymer feedstocks (powders, pellets, or compounded blends) are heated and shaped using a die or mold [88]. The principal advantage is the high level of process robustness and compatibility with established manufacturing infrastructure. However, producing a highly porous, interconnected scaffold typically requires integration with a pore-forming concept (e.g., porogen leaching, foaming, or particle-based sintering), because a purely melt-molded construct is generally dense [89]. Specifically, the melt molding technique has been explicitly demonstrated to require a combination with pore-generating strategies when porous regenerative scaffolds are targeted to be produced.

A particular engineering concern is the thermal nature of these processes, since polymers exhibit low thermal conductivity, and many biodegradable polyesters display limited thermal stability under extended residence times [90]. Consequently, local temperature gradients and slow cooling can induce chain scission, undesired crystallization changes, or property changes. These melt-processing challenges have been directly discussed as quality risks for biopolymers in melt molding contexts.

#### 4.1.2. Melt Extrusion Compounding and Filament Manufacturing for Scaffold Production

The melt extrusion method mostly serves two complementary roles for scaffold production. The first is compounding and homogenization. Twin-screw extrusion enables dispersion of bioactive fillers (e.g., hydroxyapatite, bioactive glass) and rheology modifiers, providing a route to reproducible composite feedstocks. The second is feedstock shaping. Extrusion can produce pellets or filaments suitable for subsequent forming steps (injection molding, compression molding, or material-extrusion 3D printing). A practical demonstration of this workflow is solvent-free fabrication of composite filaments for material-extrusion printing. For example, PCL/HA filaments produced via thermal extrusion have been used to print scaffolds with interconnected architectures; in such systems, increasing ceramic content can alter processing temperatures and mechanical properties, while also increasing surface roughness and hydrophilicity [91]. For example, Cossé et al. developed PLGA implants by utilizing 5- and 9-mm mini-scale twin screw extruders of the hot melt extrusion method [92]. In the study, the researchers aimed to construct sustained protein release (BSA) by matrix erosion behavior. The obtained results revealed that BSA-loaded implants released 17.0% glycolic and 5.9% lactic acid after 14 days of preparation. On the other hand, the burst release was detected as 3.7% for BSA, which also sustained protein release starting from the fourth week. Stress conditions were mimicked at 30 °C and 75% rH, which revealed a shift of erosion onset of 1 week.

#### 4.1.3. Material-Extrusion Additive Manufacturing for Architected Porous Scaffolds

**Figure 7:**
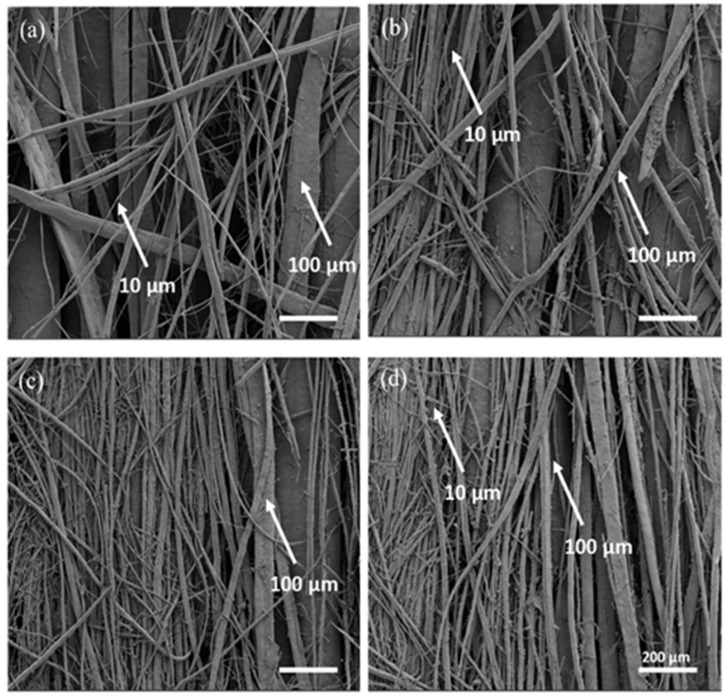
SEM images of produced microfibrous scaffolds, which were recorded at 100× magnification for (**a**,**b**) for 50/50 PCL/PEG, (**c**,**d**) for 40/60 PCL/PEG [97]. Increased fidelity from a to d.

Material extrusion is widely used for biodegradable polymer scaffold fabrication because it provides deterministic control of macro-architecture (strand spacing, laydown pattern, layer height, pore size at the printed scale) [93]. In a representative study, material extrusion is described as the most common 3D printing method, in which a prefabricated filament is deposited layer by layer, and the authors further note that it is a scalable, solvent-free route that enables controlled filler distribution in composite filaments [94]. Nevertheless, for tissue engineering scaffolds, melt-based printing imposes several constraints that must be explicitly managed in the manufacturing design space. Rheology and resolution coupling are important parameters to be considered. Higher melt viscosity improves shape fidelity but can limit nozzle flow, raise processing temperatures, and increase the risk of shear-induced degradation [95]. Thermal history, on the other hand, crucially affects cooling rate, crystallization kinetics, and thermal gradients, which influence surface and bulk properties [96]. In melt-based thermoplastic scaffolds, crystallization control has been shown to modulate surface mechanics/roughness, which can in turn influence cell responses. Bioactive integration limits: Thermolabile agents (proteins, peptides, certain antibiotics) may lose activity if exposed to melt-processing temperatures or extended residence times; this challenge becomes particularly challenging when “medicated scaffolds” are targeted. For example, Spedicati et al. reported a 3D microfibrous scaffold composed of PCL/PEG blends to reconstruct skeletal muscle tissue by benefiting from the melt-extrusion additive manufacturing method [97]. While they used PCL as the bulk material and PEG as the porogen, they obtained a micrometric, anisotropic fibrous structure with fiber diameters ranging from 10 to 100 µm in the 40/60 PCL/PEG formulation, as it was shown in Figure 7 with different formulation outputs of scanning electron microscope (SEM) imaging.

The best optimized scaffold (40/60 PCL/PEG) was used in an in vitro study with C2C12 cells, which monitored the attachment, alignment, and myotube formation for a 14-day culture period. The results revealed that anisotropic scaffold morphology enhanced the proliferation of C2C12 cells to provide skeletal muscle tissue-like conformation and orientation. Beyond *in vitro* performance, recent animal studies support the translational promise of solvent-free and additively manufactured biodegradable scaffolds. In a rabbit segmental femoral defect model, 3D-printed PCL/nHAp implants showed tissue ingrowth, new bone formation, and biomechanical healing comparable to autograft at early follow-up, while also underscoring the relevance of patient-specific design. Likewise, a recent low-temperature, solvent-free in situ printing platform for PCL/HA constructs demonstrated robust new bone formation in a critical-sized defect model, highlighting the possibility of personalized, point-of-care scaffold delivery [98].

#### 4.1.4. Hybrid Melt-Based Routes: Microcellular Injection Molding and Sacrificial Leaching

A major opportunity for melt-based routes lies in hybridization, combining high-throughput shaping (e.g., injection molding) with controlled pore formation. Microcellular injection molding (MIM) exemplifies this strategy, where a physical blowing agent generates micro-porosity during molding, and a secondary sacrificial phase can be leached to increase openness and interconnectivity [99]. An illustrative example is the fabrication of fibrillated, interconnected porous PCL scaffolds via blending PCL with water-soluble PEO as a sacrificial material, followed by microcellular injection molding and leaching. This approach achieved high porosity (reported up to ~89.5% for certain blend ratios) and improved pore interconnectivity [100]. Key process parameters to report (and later use for comparison across solvent-free approaches) include barrel/nozzle temperatures, mold temperature, gas dosage and pressure, residence time, depressurization/foaming dynamics, porogen fraction and particle size, and leaching conditions (time and fluid exchange frequency). These variables strongly govern hierarchical porosity, interconnectivity, and mechanical integrity. For instance, Huang et al. developed PCL scaffolds by blending them with PEO as a sacrificial material to provide desired porosity [101]. The study was conducted by optimizing the PCL/PEO ratios, which a 50% PCL/50% PEO blend provided the highest porosity up to 89.5%. The selected scaffold was evaluated in vitro by utilizing 3T3 fibroblast cells, which demonstrated high cell viability thanks to the formation of elongated and spindle-shaped pore structures and high biocompatibility.

### 4.2. Solid-State and Low-Temperature Solvent-Free Methods

While melt-based routes are attractive for scalability, they can be restrictive for thermally sensitive polymers and bioactive needs. Consequently, solvent-free methods that operate at solid-state or near-ambient temperatures provide valuable alternatives. These include solid-state foaming combined with benign porogen extraction, solid-particle consolidation and sintering (including CO_2_-assisted sintering of microspheres), and laser-driven sintering of polymer powders.

#### 4.2.1. Immiscible Blending and Benign Porogen Extraction with Solid-State Foaming

A particularly powerful low-solvent-burden concept is immiscible polymer/porogen blending to form co-continuous structures, followed by extraction of the sacrificial phase using benign liquids (often water), optionally coupled with solid-state foaming to create hierarchical porosity. A fully organic-solvent-free example uses PLA–sucrose blends prepared by extrusion mixing, followed by solid-state CO_2_ foaming and water leaching of sucrose. This approach produced scaffolds with >90% porosity and tunable pore sizes (reported in the ~25–200 μm range), with pore size and scaffold strength modulated by extrusion temperature and foaming parameters [102]. The study explicitly motivates the method as a solvent-free route that avoids concerns about residual organic solvents affecting cell growth. From a manufacturing standpoint, this route is conceptually appealing because it decouples (a) bulk shaping and phase morphology formation (via extrusion) from (b) pore refinement (via foaming) and (c) phase removal (via water leaching). However, the time scale associated with CO_2_ saturation in some solid-state foaming workflows (e.g., multi-day saturation) can become a throughput bottleneck unless engineered for continuous or accelerated saturation.

#### 4.2.2. Solid-State Mixing (Cryomilling) + Molding + Leaching for Improved Pore Uniformity

Uniform distribution of porogens is a frequent limitation of particulate leaching routes. Solid-state mixing, particularly cryomilling, has been proposed to improve porogen dispersion and scaffold uniformity without introducing solvents [100]. Jonnalagadda et al. describe a solventless approach in which salt particles and PEO were mixed (manually or via cryomilling) with PCL, then compression molded and water-leached, aiming to obtain interconnected porous scaffolds with uniform morphologies [103]. This topic can be positioned as an example of “process intensification” for solvent-free scaffold manufacturing: rather than adding chemical steps, the process improves the microstructural outcome by engineering the mixing physics.

#### 4.2.3. Microsphere-Based Scaffolds and Low-Temperature CO_2_ Sintering

Microsphere-based scaffolds represent a “bottom-up” solvent-free assembly strategy where microspheres themselves are the building blocks of the scaffold. A key feature of sphere packing is that the pore network can exhibit high intrinsic interconnectivity; indeed, microsphere-based scaffolds have been described as initially possessing a pore network with 100% interconnectivity due to sphere packing, and sintering then converts the packed bed into a mechanically integrated construct [104].

Low-temperature consolidation can be achieved by sub-critical CO_2_ sintering by plasticizing polymer surfaces at near-ambient temperatures. It enables adjacent microspheres to fuse while preserving cell viability. Singh et al. demonstrated that the cell viability was sustained during sub-critical CO_2_ sintering of PLG microspheres in the presence of cells at near-ambient temperatures [105]. The proposed study explicitly contrasts this with conventional heat and solvent sintering approaches that are not cytocompatible. Processing conditions described in this context included exposure to CO_2_ at sub-critical levels followed by controlled depressurization. It promoted surface swelling and interfacial chain mobility sufficient for microsphere adhesion and scaffold formation. Thus, CO_2_ sintering has several strategically important implications, including thermal gentleness relative to melt-based processing. It demonstrates potential compatibility with cell- or bioactive-loaded constructs under carefully tuned conditions. This is important for the elimination of organic solvents during scaffold assembly and scalability through parallel molds or continuous CO_2_ processing chambers.

#### 4.2.4. Powder-Based Sintering: Selective Laser Sintering (SLS)

Selective laser sintering (SLS) is a solvent-free additive manufacturing method that is utilized for the consolidation of polymer powders layer-by-layer with the aid of localized heating [106]. In the context of solvent-free scaffold manufacturing, SLS is deemed to use a CO_2_ laser to raise the temperature above Tg to induce the coalescence of adjacent particles. Despite their unique solvent-free nature and potential for complex geometries, biodegradable polymers suffer from some limitations, such as material availability and process–property relationships. Santos et al. reported a study of solvent-free techniques for the processing of designed scaffolds for utilization in regenerative medicine, where SLS is noted as suffering from a limited choice of materials relative to material extrusion approaches [82].

### 4.3. Advantages and Limitations of Solvent-Free Processing

Solvent-free production of the scaffolds offers superior translational advantages. However, it is not universally optimal; rather, it defines a manufacturing design space with distinct trade-offs. Solvent-free scaffold production evades the inclusion of organic solvents that may damage biocompatibility. In terms of drug-loaded scaffolds, solvent-free processing has been reported as preferable because solvent-based leaching/extraction steps can cause dramatic loss of loaded agents and introduce additional residue risks [107] On the other hand, melt molding technologies have been demonstrated as scalable, reproducible, and cost-effective, where broadly used for commercial polymer processing. However, their translation to porous scaffolds requires deliberate pore-forming strategies, since it does not naturally contain them [108]. Material extrusion is widely utilized because it enables controllable internal architecture. Moreover, it is suitable as a scalable and solvent-free method for patient-specific devices and composite scaffolds. Solvent-free methods can improve loading yields for certain bioactive agents by avoiding washing out during solvent-based post-processing. Thus, this motivation has been highlighted in prior solvent-free scaffold processing analyses.

On the other hand, solvent-free processing also suffers from some limitations. Melt-based methods inherently impose thermal and shear histories. Quality issues can arise because of poor thermal conductivity and limited thermal stability of polymers, which may lead to partial degradation [68]. Moreover, thermally sensitive bioactive agents may lose activity during melt-based processing. Many melt-based processes do not naturally yield the high porosity and interconnectivity demanded for cell infiltration. Therefore, it motivates hybrid routes such as foaming, porogen leaching, and sacrificial phases. While effective, hybridization can increase step count and introduce leaching-associated losses for drug-loaded constructs. For semi-crystalline thermoplastics, crystallinity and crystal size distributions depend on thermal history and can influence surface characteristics relevant to cell fate [109]. This creates both an opportunity (tunable properties) and a challenge (manufacturing variability) for melt-based scaffolds. Solid-state foaming and CO_2_ saturation steps can be time-intensive, potentially limiting manufacturing throughput unless redesigned for faster mass transfer and continuous operation. Table 1 shows the comparison of solvent-free techniques.

### 4.4. Scale-Up, Cost, and Manufacturing Throughput

Scale-up of solvent-free scaffold manufacturing is not solely an equipment issue. Rather, it is an integrated problem spanning feedstock preparation, process control, post-processing, and quality assurance. For industrial translation, a solvent-free route is required to deliver geometry and porosity within specification, consistent molecular weight/crystallinity, acceptable residue and extractables profiles, and economically viable throughput.

#### 4.4.1. Scale-Up Trajectories by Process Family

Melt molding routes are among the most industrially established and are frequently described as scalable and reproducible. Their throughput potential is high, but scaffold-specific porosity requirements usually necessitate additional steps (foaming, leaching, or sacrificial phases), which can reduce the effective line throughput and introduce additional validation requirements. Material-extrusion additive manufacturing is positioned as a scalable, solvent-free method and constitutes a major fraction of the 3D printing landscape [114]. Furthermore, it supports complex and patient-specific architectures but remains inherently toolpath-limited, meaning throughput scales with deposition rate and build volume. Practical scale-up strategies include multi-nozzle printing, parallelization (printer farms), higher-throughput pellet-fed extrusion heads, and hybrid workflows where printing defines macro-architecture followed by high-throughput post-processing (e.g., foaming or impregnation). Microcellular injection molding and hybrid molding/leaching workflows are particularly promising for scale because injection molding is intrinsically a high-volume manufacturing platform [115]. Huang et al. reported in a PCL/PEO approach, in which the authors explicitly describe the combined method as practical, eco-friendly, and suitable for mass production of scaffolds with desired geometries [101]. However, if leaching is required, that step can dominate cycle time and facility footprint (tanks, water handling, and drying), and must be addressed with engineered leaching kinetics, closed-loop water systems, and in-line weight/porosity endpoints. Solid-state foaming and immiscible blending approaches offer strong sustainability and safety advantages (no organic solvents) and deliver high porosity with tunable pore sizes; yet saturation/foaming logistics can be throughput-limiting in batch mode. For industrial translation, this motivates engineering development toward intensified CO_2_ transport (thin sections, continuous saturation, and elevated diffusion driving forces within safe limits) and inline monitoring of CO_2_ uptake and foam morphology. Microsphere-based sintering and CO_2_-assisted consolidation can be attractive where controlled release, gradients, and high interconnectivity are central requirements [116]. Importantly, microsphere fabrication and sintering can be “reasonably simple, inexpensive, and in many instances scalable” in principle, but the overall cost is highly sensitive to microsphere production yield, size distribution control, and sintering cycle times. Sub-critical CO_2_ sintering additionally opens routes to low-temperature consolidation and potentially cell-compatible processing under tuned conditions, which may reduce downstream costs associated with post-fabrication seeding steps.

#### 4.4.2. Dominant Cost Drivers and Practical Cost-Containment Levers

Across solvent-free scaffold routes, the dominant cost drivers typically include feedstock costs, energy intensity, cycle time, yield, and quality control overhead. In feedstock costs, the polymer grade, molecular weight distribution requirements, composite fillers (e.g., HA), and porogens should be considered. In energy intensity, heating (melt routes), pressurization (CO_2_ saturation/foaming/sintering), and drying after leaching should be evaluated. In cycle time and yield, long saturation/leaching times and scrap from warped or dimensionally off-spec parts should be analyzed. In quality control overhead, micro-CT/porosimetry, mechanical testing, molecular weight tracking, and batch traceability should be monitored. In this context, solvent-free processing can reduce costs by eliminating solvent handling and recovery, but only if the process is engineered to avoid replacing “solvent burden” with “time burden” (e.g., very long leaching or saturation steps).

#### 4.4.3. Economic Challenges and Risk Management for Scale-Up and Clinical Use

While nominal scaffolding throughput is important, when evaluating the economic viability of scaling up scaffolding on a larger scale, a more comprehensive cost-of-goods framework is needed, including medical-grade feedstock; controlled manufacturing environment; sterilization; packaging; validation of the manufacturing process; testing for rejection; providing traceability; and considering the costs associated with scrap, rework, or failures by batch. This framework can be particularly burdensome for patient-specific or additively manufactured scaffolding because current regulations require that we test for characteristics that are specific to the manufacturing process, not just based on reproducing geometry. Additionally, in the case of biodegradable polymers overall, many recent studies show that feedstock costs, energy consumption, and downstream recovery or purification of biodegradable polymers are still significant barriers to commercialization. Therefore, developing a business case for scaffold translation should be based on technoeconomic rather than basic technical needs. To this end, when scaling up, the focus of the manufacturing process should be to achieve high first-pass yields, reduce post-processing, establish robust raw-material specifications, and select manufacturing routes to reduce the cost of nonconformance while ensuring that the scaffold has the mechanical properties and reproducibility necessary for sustaining life [117].

As a lifecycle activity rather than as a final product release step, risk management will also continue to expand. The ISO 14971:2019 Privacy of Individual Data (PID) Standard provides a framework for manufacturing a product from conception through disposal of the prototype that will systematically identify the hazards associated with the manufacture of a product and then estimate and evaluate the risk associated with those hazards; implement controls to eliminate or reduce the risk and monitor the effectiveness of those controls throughout the entire lifecycle of the product. Likewise, the EU MDR stipulates a continuous and iterative process of managing all aspects of risk that include the identification of risk and the determination of the appropriate benefits to be achieved from the design of the device and documentation of those benefits in the technical file for the device. The FDA’s QMSR now aligns the U.S. device quality system requirements with ISO 13485 and also requires that risks be controlled and managed throughout the design and lifecycle of the device.

The manufacturing risks associated with biodegradable scaffolds can be classified into two main categories: those associated with batch-to-batch variations in pore architecture, fiber diameters, or interconnectivity, and the thermal processing of the scaffold, as well as those due to residual porogens, extractables, contamination, dimensional inaccuracies, and loss of bioactive agent potency. The use-related risks of biodegradable scaffolds include mechanical failure of the scaffold after implantation, infection as a result of inadequate sterilization or handling, adverse local responses to degradation products, uncontrolled release kinetics from the scaffold, and a mismatch between resorption of the scaffold and tissue regeneration. Thus, when manufacturing biodegradable scaffolds using green technology, manufacturers should frame their efforts not only around sustainability, throughput, and cost-effectiveness, but also around a framework of controlled reproducibility of their products, validated process windows, and the use of clinically relevant acceptance criteria [118].

## 5. Supercritical CO_2_-Based Scaffold Manufacturing

Supercritical carbon dioxide (scCO_2_) has emerged as a particularly compelling platform for green, solvent-minimized scaffold manufacturing. This is mostly because it can act simultaneously as a physical blowing agent for pore generation, a polymer plasticizer that enables processing at reduced temperatures, and a solvent/diffusion enhancer for loading small molecules into polymer matrices [83,119,120]. In tissue engineering, CO_2_-based processing gained early attention for producing highly porous foams, but adoption was historically limited by challenges such as pore interconnectivity and the formation of a dense non-porous “skin layer”. Subsequent dedicated studies clarified how to tune saturation, venting, and thermal histories to mitigate these limitations, renewing interest in scCO_2_ as a practical manufacturing tool [92,93]. Within the “green and scalable manufacturing” framing, scCO_2_ processing is especially valuable because it can support integrated manufacturing workflows, where scaffold architecture formation is coupled with drug loading and/or terminal sterilization in a single high-pressure platform, an approach that directly addresses translational requirements for reproducibility, safety, and process intensification.

### 5.1. Fundamentals of Supercritical CO_2_ Technology

A supercritical fluid is a dense-phase fluid maintained above its critical temperature and pressure, where the distinction between liquid and gas disappears. For CO_2_, the critical point is close to ambient conditions (Tc ≈ 31.1 °C; Pc ≈ 73.8 bar), which enables processing under relatively mild temperatures compared with many polymer manufacturing routes [121].

#### 5.1.1. Why scCO_2_ Is Attractive for Biomaterial Manufacturing

The appeal of scCO_2_ in scaffold manufacturing is rooted in its hybrid transport/solvation properties. Above the critical point, CO_2_ exhibits liquid-like density (supporting solvent power) combined with gas-like viscosity and diffusivity, supporting rapid mass transport [122]. These properties are also tunable via pressure and temperature, providing a controllable process space compared with conventional solvents whose properties are far less pressure dependent.

From a sustainability and regulatory perspective, scCO_2_ is frequently highlighted as inexpensive, non-toxic, and non-flammable, and it can be removed from the product by simple depressurization; CO_2_ can also be regenerated and recycled within closed-loop equipment architectures. For example, Pantić et al. reported a polycaprolactone-chitosan-based scaffold by utilizing supercritical carbon dioxide [123]. In the study, researchers produced PCL foams and chitosan aerogel beads to provide a drug carrier platform for tissue engineering [123]. The analyzed results demonstrated that the integration of mesoporous chitosan aerogels into the macroporous structure of PCL caused a dramatic change in the textural, morphological, and thermal properties of the initial foams. The contrast behavior was detected in the scaffolds, which showed a mesoporous structure unlike foams. Thus, they increased the content of the aerogels in the formulation to obtain larger specific surface areas. The condition optimization provided different swelling behavior, degradation, and distinct release profiles, which provided crucial output for molecule-releasing scaffold studies.

#### 5.1.2. Polymer-CO_2_ Interactions: Sorption, Swelling, and Plasticization

The core enabling phenomenon for scaffold processing is the ability of CO_2_ to sorb into many polymers, inducing swelling and plasticization [124]. At the molecular level, dissolved CO_2_ increases chain mobility and lowers resistance to chain rotation, which depresses Tg and can reduce effective Tm and melt viscosity in semicrystalline systems. Importantly, CO_2_ sorption is polymer-specific and depends on specific parameters, including chemical functionality and intermolecular interactions, morphology, and free volume, which influence diffusivity and solubility, and copolymer composition. These dependencies translate into real manufacturing outcomes in foaming efficiency, pore stability, skin formation, and drug-loading capacity, making polymer grade selection and material history in thermal treatment and crystallinity critical design variables.

#### 5.1.3. Solubility Limitations and Implications for Polymer Selection

A key obstacle to developing a viable method for producing scaffolds using scCO_2_ is the fact that, at the temperature and pressure used to produce scCO_2_, scCO_2_ cannot dissolve most high-molecular-mass polymers. In terms of dissolving polymers, only a limited subset of polymers can be dissolved in scCO_2_, while in many situations the process of dissolving CO_2_ into the polymer is far more important than dissolving the polymer into CO_2_. Recent analyses have shown that the vast majority of high-molar-mass polymers can only be completely dissolved in scCO_2_ at very low temperatures, and only a few specific classes of polymers (e.g., ionic) exhibit good levels of solvation in scCO_2_ at moderate temperatures. Additionally, most polymers that are used to make tissue scaffolds are expected to dissolve only partially in scCO_2_ and exhibit other characteristics, such as swelling or softening, instead of completely dissolving [125]. Even for the same types of products made from these types of polymers, many times, significant differences are found within these families of products. For example, certain types of silicone elastomers and poly(L-lactide-co-ε-caprolactone) have been shown to cause considerable swelling when in combination with CO_2_, while most polyolefins and low-density polyethylene (LDPE)-coated polyester do not solvate as well. Thus, it is clear that the amount of chain motion available to the polymer, the amount of free volume in the polymer, and the amount of amine (CO_2_-attracting) functional groups in the polymer are some of the key considerations related to how well a polymer will dissolve in scCO_2_ [126].

Due to this limitation, there are many practical implications for the manufacturing of scaffolds. First and foremost, the majority of applications of scCO_2_ in the field of tissue engineering rely on either swelling, softening, foaming, impregnating, extracting, or sterilizing the scaffold polymer, rather than on the ability of scCO_2_ to produce a homogeneous solution of the scaffold polymer [127]. Second, the extent of the usable “material space” is greatly reduced for systems that are highly crystalline in nature, have low volumes of free volume, or contain a lot of fillers. For example, increasing the amount of filler material (e.g., hydroxyapatite) in a poly(L-lactide) (PLLA)-based composite has been shown to decrease the amount of CO_2_ that can be dissolved in the PLLA composite. Finally, the limited solubility of many polymers inherently leads to the inclusion of additional monomers, less amorphous grades, and CO_2_-compatible functional groups being suggested as better alternatives when developing polymer scaffolds requiring stronger interactions between CO_2_ and the polymer. Therefore, it can be concluded that scCO_2_ can more appropriately be viewed as a green solution for the processing of polymers, rather than as a universal green polymer solution, given that the potential applications of scaffolds developed from scCO_2_ depend on the selection of polymers to be processed, their structures, functional groups, and targeted processing unit operations [128].

#### 5.1.4. Generic Process Steps and Controllable Knobs

Across foaming and impregnation, most scCO_2_ processes can be described by a common sequence, which is pressurization and saturation, soaking/diffusion, and depressurization. In pressurization and saturation, the polymer is exposed to scCO_2_ at controlled temperature and pressure until sufficient sorption/swelling is achieved. In the soaking/diffusion period, the system is held to approach sorption equilibrium and allow CO_2_ and solute, in loading operations, to diffuse within the matrix. In depressurization, also known as venting, pressure is reduced in a controlled manner; CO_2_ solubility drops, nucleation occurs, and pores grow and/or solute precipitates. A central design principle is that the venting/depressurization rate competes with diffusion-driven pore growth. Rapid venting can yield many nucleation sites and more uniform, smaller pores, whereas slower venting provides additional time for diffusion and can produce larger pores and broader distributions [129].

### 5.2. scCO_2_ Foaming of Biodegradable Polymers

**Figure 8:**
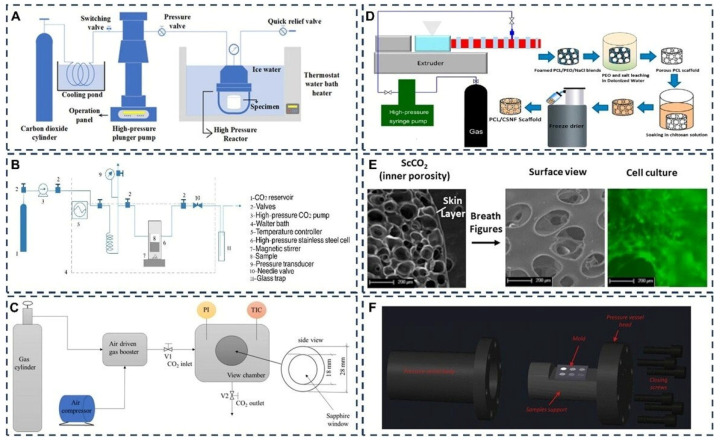
scCO_2_ foaming technology utilized by particle leaching integration (**A**), illustration of SFM technology (**B**), scCO_2_ foaming method integrated with impregnation technique (**C**), scCO_2_ foaming technology in combination with freeze-drying (**D**), ssCO_2_ providing bubble structure by utilization of BFs57 (**E**), the patterning system composed of mold patterning and scCO_2_ foam technology (**F**) copyright © 2019 Elsevier B.V [15].

scCO_2_ foaming is a solvent-free route to generate porous architectures in biodegradable polymers, often positioned as an alternative to solvent casting/particulate leaching or other solvent-intensive processes. It is frequently described as operating under mild conditions while avoiding organic solvents, an advantage that becomes particularly important when scaffolds are intended to carry drugs or bioactives. Figure 8 shows the general methods of scCO_2_ foaming techniques.

#### 5.2.1. Solid-State vs. Melt-State scCO_2_ Foaming

A widely used conceptual distinction is whether foaming occurs in the solid state as near/just above Tg, diffusion and chain mobility are limited; pore growth can be constrained, potentially increasing closed porosity. In the melt state, as near/above Tm for semicrystalline polyesters, higher chain mobility can enable larger pores and higher expansion but may increase the risk of pore collapse unless melt strength and crystallization kinetics provide adequate stabilization [130]. For example, in PCL systems, the transition from solid-state to melt-state foaming with increasing temperature has been associated with larger pore size and higher porosity, while also emphasizing the importance of intrinsic polymer properties (rheology and crystallization) for maintaining stable interconnected macropores. In a study conducted by Zhang et al., porous polyglycolic acid (PGA) scaffolds were reported by utilizing the melt-foaming technique using ssCO_2_. They conducted a Haake rheometer study on PGA before the design of the foaming strategy in order to observe the effect of scCO2 on PGA, which was executed by high-pressure differential scanning calorimetry (DSC) [131]. The obtained results exhibited that the elasticity and viscosity of the polymer could be dramatically enhanced by adjusting the temperature to ensure the growth of bubbles at the initial depressurization. The dissolution of compressed CO_2_ was injected into the PGA melt, and the following depressurization process at a relatively low temperature with high PGA melt strength provided a controllable porosity from 39% to 74%, obtaining average pore sizes ranging from 5 to 50 μm.

scCO_2_ foaming studies should be compared using a unified set of controllable parameters and measurable outputs. In terms of saturation pressure (P) and temperature (T), setting CO_2_ density, sorption capacity, and plasticization strength is required. In soaking time (t_soak_), administering how deeply CO_2_ penetrates should be adjusted; insufficient soaking can yield a non-porous core with a porous shell, reflecting mass-transfer limitations. The depressurization rate (dP/dt) and pressure profile, as single-step vs multi-step, strongly impact nucleation density, pore growth time, and pore interconnectivity. On the other hand, cooling rate and thermal history influence crystallization-driven locking-in of pore structures and final morphology. A particularly important nuance for biodegradable polyesters is that intrinsic polymer rheology and crystallization kinetics can be decisive. Since higher melt strength can hinder pore expansion and fracture and reduce interconnectivity, while rapid crystallization can stabilize pores and reduce collapse, this means that the “same conditions” may produce very different scaffold morphologies across molecular weights and grades [132].

#### 5.2.2. Persistent Morphological Challenges: Skin Layer and Interconnectivity

Two widely reported scCO_2_ foaming issues are a dense, non-porous skin layer and pore interconnectivity. In the non-porous skin layer, early CO_2_-foamed polyester scaffolds showed a non-porous surface layer and low pore interconnectivity, in part because CO_2_ rapidly escapes from the sample edges during depressurization [133]. This layer is undesirable because it can impede cell infiltration and transport; one pragmatic mitigation strategy has been post-processing removal, which should also be discussed explicitly in laboratory-scale scCO_2_ scaffold fabrication workflows. In pore interconnectivity, it can be improved by carefully tuning depressurization, soaking time, and cooling dynamics. For instance, controlling the depressurization rate has been reported as a lever for pore size and interconnectivity control in multiple systems [134]. However, achieving high interconnectivity by “pure” foaming alone remains non-trivial for certain polymers (notably low-melt-strength systems), motivating hybridization strategies.

#### 5.2.3. Composite Scaffolds and the Role of Fillers

scCO_2_ foaming can be extended to composites such as PLA/ceramic fillers. However, fillers can alter nucleation, CO_2_ sorption, and pore-wall stability. In reported datasets, filler loading has been described as constrained by the requirement to preserve adequate pore size and interconnections. Specifically, some composite foams show more heterogeneous or closed pore architectures depending on filler type and concentration. Preclinical evidence is also emerging for scCO_2_-processed scaffolds. A hierarchically porous PLA/PBS-based scaffold fabricated by melt blending and supercritical CO_2_ foaming promoted substantial new bone formation, robust angiogenesis, and lamellar bone maturation in a rat calvarial defect model, without apparent systemic toxicity. These findings suggest that scCO_2_-enabled scaffold fabrication may offer not only environmental and processing advantages, but also biologically meaningful in vivo performance [133].

### 5.3. scCO_2_-Assisted Drug and Growth Factor Loading

Beyond pore formation, scCO_2_ enables post-fabrication functionalization of biodegradable polymer scaffolds through impregnation/deposition of drugs and selected bioactive molecules. This is strategically important for tissue engineering translation because it can reduce solvent exposure, simplify purification, and enable multifunctional scaffolds.

#### 5.3.1. scCO_2_-Assisted Impregnation/Deposition: Principle and Clinical Relevance

scCO_2_-assisted impregnation has been widely discussed as a method to load active pharmaceutical ingredients into polymeric implants, with particular advantages including mild temperatures suitable for thermosensitive drugs and recovery of a final device free of solvent residues [135]. In particular, scCO_2_ can dissolve or partially solubilize the active ingredient depending on solubility constraints, transport it to the polymer surface, enhance diffusion by plasticizing/swelling the polymer, and induce precipitation and entrapment upon depressurization. Therefore, the following factors should be explicitly compared across studies. First, drug solubility in scCO_2_ is often limited for many drugs, even at elevated pressure, which can be addressed via pressure/temperature optimization or cosolvent strategies in certain cases [136]. Second, polymer swelling and diffusivity, polymer chemistry, crystallinity, and free volume determine how deeply the solute can penetrate. Third, depressurization protocol influences whether loading is dominated by bulk impregnation versus surface deposition/crystallization. Fourth, for coupled morphology changes, where CO_2_ absorption/desorption can induce foaming, aggregation, or crystallinity shifts, those effects that may be either beneficial in added porosity or detrimental. These determinants should be mapped to functional outcomes: loading mass fraction, release kinetics as burst vs. sustained, and preservation of scaffold mechanics and degradation behavior.

#### 5.3.2. Growth Factors: Opportunities and Constraints

Many growth factors and proteins have low solubility in scCO_2_, so direct impregnation may be challenging; nevertheless, scCO_2_ can still play a key role in hybrid constructs where bioactive proteins are pre-encapsulated (e.g., within polymer phases or microparticles) and incorporated during CO_2_ processing [137]. A representative example described in a tissue-engineering-focused CO_2_ processing review involves a construct where VEGF and BMP-2 were incorporated into a hybrid scaffold system processed using supercritical CO_2_; such reports illustrate the conceptual viability of coupling CO_2_ processing with multi-signal delivery designs, including in vivo regeneration outcomes [138].

### 5.4. Hybrid scCO_2_ Processing Strategies

A central advanced manufacturing narrative is that scCO_2_ is rarely used in isolation in translational workflows. Rather, it is increasingly integrated with other fabrication steps to overcome limitations of stand-alone foaming or impregnation and to enable multifunctionality.

#### 5.4.1. Foaming + Templating/Leaching for Enhanced Interconnectivity and Gradients

Hybridization with porogens or particulate templating can mitigate skin-layer and interconnectivity issues. In the CO_2_ processing literature, microparticulate templating, like NaCl gradients, has been used to create open-pore foams with spatial gradients in porosity and pore size. Such strategies are highly relevant to tissue engineering, where gradients like bone–cartilage, tendon–bone, and osteochondral can be functionally advantageous [139].

#### 5.4.2. scCO_2_ Foaming as Post-Processing for Additively Manufactured Scaffolds

A promising hybrid paradigm is macro-architecture by 3D printing followed by micro-/nano-porosity by gas/scCO_2_ foaming, yielding hierarchical scaffolds that combine deterministic large pores (100–800 µm) with smaller micropores (1–10 µm) [140]. This concept is particularly compatible with the broader scope of our review (solvent-free melt processing and MEW) because scCO_2_ can act as a low-chemical-burden post-treatment to introduce secondary porosity and/or to enable subsequent impregnation.

#### 5.4.3. One-Pot Foaming + Sterilization and the “Terminal Processing” Concept

One of the most translationally compelling hybrid strategies is scCO_2_-driven one-pot scaffold preparation and sterilization. A reported protocol applied scCO_2_ to prepare and sterilize PCL/PLGA scaffolds, with sterilization conditions screened against Gram-positive/Gram-negative bacteria and spores, targeting stringent sterility assurance, for which SAL 10^−6^ terminology is used in the work [141]. This is important because terminal sterilization remains a major barrier for advanced polymer scaffolds. ScCO_2_ sterilization has been reported as less compromising to mechanical/rheological properties in sensitive biomaterials when compared with some established sterilization methods, including gamma irradiation, ethylene oxide, steam, and is increasingly positioned as a green alternative able to meet regulatory sterility expectations while preserving material integrity.

### 5.5. Scalability, Equipment Design, and Industrial Feasibility

Industrial feasibility of scCO_2_ scaffold manufacturing depends on both equipment architecture, such as the pressure vessel and CO_2_ handling, and process design choices that determine throughput, reproducibility, and the cost of goods.

#### 5.5.1. Equipment Building Blocks for scCO_2_ Foaming and Loading

A scCO_2_ processing unit typically includes a high-pressure processing vessel (autoclave) where the polymer is in contact with scCO_2_. Moreover, a pump/compressor is used to reach and maintain pressure, temperature control in heating jackets, and heat exchangers. Finally, a back-pressure regulator/valve is used to control depressurization profile, optional recirculation and conditioning modules for CO_2_ handling, and solvent/solute management. For impregnation specifically, the basic architecture consists of the loading chamber plus a system for handling and conditioning the supercritical solvent, with progressive scale-up from lab to pilot and industrial units [142].

#### 5.5.2. Throughput Pathways: Batch vs. Continuous Processing

A critical scale-up decision concerns whether scaffolds are processed in batch or continuous mode. Batch foaming is widely used in research because it allows fine control of parameters and is relatively simple [143]. However, extrusion foaming is often described as the industrially preferred route for foaming due to continuous production and easier scale-up potential. For tissue engineering translation, batch scCO_2_ processing may remain attractive for high-value, lower-volume products (patient-specific scaffolds and combination products), whereas continuous approaches may be required for high-volume standardized implants.

#### 5.5.3. Scale-Up Constraints Unique to Porous Scaffolds

Unlike many commodity foams, tissue scaffolds must meet stringent requirements, including porosity, interconnectivity, mechanical performance, biocompatibility, and sterilization. The most scale-sensitive constraints include crucial parameters. The first one can be considered mass transfer limitations in thick parts. Insufficient soaking can produce a non-porous core, implying that diffusion times increase sharply with part thickness. The second one is deemed skin-layer control. Dense surface layers arise from rapid CO_2_ escape at the surface during depressurization and can act as a barrier to cell colonization and factor delivery; manual removal is commonly used at the lab scale but is undesirable industrially, motivating engineering controls [144]. Finally, reproducibility of pore interconnectivity is another crucial parameter, since depressurization rate and thermal history must be controlled tightly to avoid non-uniform pore structures.

#### 5.5.4. Cost, Sustainability, and Operational Considerations

While scCO_2_ is often framed as a green solvent, industrial feasibility still hinges on capital expenditure for high-pressure equipment (which can be significant), CO_2_ recycling and closed-loop operation, which reduces emissions and operating cost, and validation and quality systems, especially for drug-loaded combination products and terminally sterilized scaffolds [145]. Notably, scCO_2_ sterilization is increasingly positioned as a technology that can achieve regulatory-relevant sterility without altering the properties of sensitive materials, an argument that can strengthen industrial adoption when traditional methods compromise polymer integrity.

## 6. Melt Electrowriting (MEW) as a Green Precision Manufacturing Tool

Melt electrowriting (MEW) is an electrohydrodynamic (EHD) additive manufacturing technique that enables the direct writing of continuous thermoplastic microfibers into highly ordered layer-by-layer architectures [146]. In contrast to solution electrospinning, MEW relies on polymer melt extrusion and fiber solidification by cooling, thereby avoiding organic solvents, solvent evaporation, and the associated need to demonstrate the absence of harmful residuals in the final construct, an inherently green advantage when designing clinically relevant biodegradable scaffolds. From a tissue-engineering standpoint, MEW uniquely bridges the gap between micro-/nanofibrous systems that mimic extracellular matrix (ECM) features and deterministic scaffold architectures typical of additive manufacturing [18]. It provides programmable control over microfiber placement, enabling architectures that guides cell alignment and neo-tissue organization across multiple layers and, increasingly, onto complex collectors and anatomically relevant surfaces.

### 6.1. Fundamental Principles of Melt Electrowriting

Essentially, MEW combines thermally controlled melt extrusion with electric-field-driven jet formation and computer-controlled fiber deposition. A polymer melt is extruded through a nozzle, though filament-based systems are also emerging. An electric potential at the nozzle induces the formation of a Taylor cone from which a stable jet is drawn toward an oppositely charged/grounded collector. Coordinated motion of the collector and/or print head enables deposition along defined tool paths, and repeated stacking produces three-dimensional (3D) scaffolds. A useful engineering framing is to view MEW as a sequence of coupled stages, which are heating and extrusion, Taylor cone formation, jet formation/transport, and jet deposition, because each stage imposes distinct constraints on stability, fiber diameter, and placement fidelity [147].

MEW is intrinsically multi-parametric, and stable printing emerges from balancing melt rheology, electrostatic forces, and kinematic conditions. The most influential controllable parameters typically include melt temperature, applied voltage, pressure/flow or feed rate, collector speed with toolpath acceleration, and working distance in the nozzle–collector gap [148]. Two concepts are particularly important for interpreting MEW studies and for designing reproducible protocols. The first is jet lag and flight-path effects. Because the jet travels along a catenary-like path, there can be a measurable offset between nozzle position and deposition location, which directly impacts geometric accuracy in sharp turns and during speed changes [149]. The second is critical translation speed (CTS). Collector speeds below the CTS can yield wave-/loop-like deposition, while speeds above the CTS can produce direct writing but with jetlag effects; operating slightly above the CTS has been used to achieve improved deposition accuracy in certain polymer systems [150].

MEW commonly yields fibers in the micrometer range, and sub-micrometer fibers have also been reported in specific setups. On the other hand, recent industry-oriented discussions describe fiber diameters spanning from sub-micrometer to tens of micrometers, depending on the hardware and operating regime [151]. Material choice is a central determinant of printability and sustainability-relevant processing windows. PCL is widely used because it is biodegradable, semicrystalline, and melts at relatively low temperatures, enabling more accessible processing and reduced thermal stress compared to higher-melting polymers [152]. However, MEW is expanding to additional biodegradable polyesters and blends, with increasing attention to how melt viscosity, conductivity, and charge accumulation influence placement fidelity and interlayer bonding.

### 6.2. Scaffold Architecture and Design Freedom

MEW’s primary differentiator is the combination of microfiber-scale resolution with deterministic fiber placement. By programming toolpaths and coordinating motion, MEW can produce lattices and patterns with controlled fiber orientation, spacing, and stacking sequences. These features are important for allowing precise tuning of pore size, anisotropy, and multi-layer architecture in ways that are difficult to reproduce with stochastic electrospun mats. Historically, MEW structures were mostly fabricated on flat collectors. More recently, design freedom has been expanded through customized collector geometries and molds and multi-axis kinematics that maintain constant working distance and favorable field orientation during printing onto curved surfaces. A notable example is the development of a five-axis MEW platform enabling collision-free printing onto complex 3D geometries and demonstrating microfibers stacked onto collectors resembling a trileaflet valve and a bifurcating vessel, an important step toward clinically relevant shapes [153].

**Figure 9:**
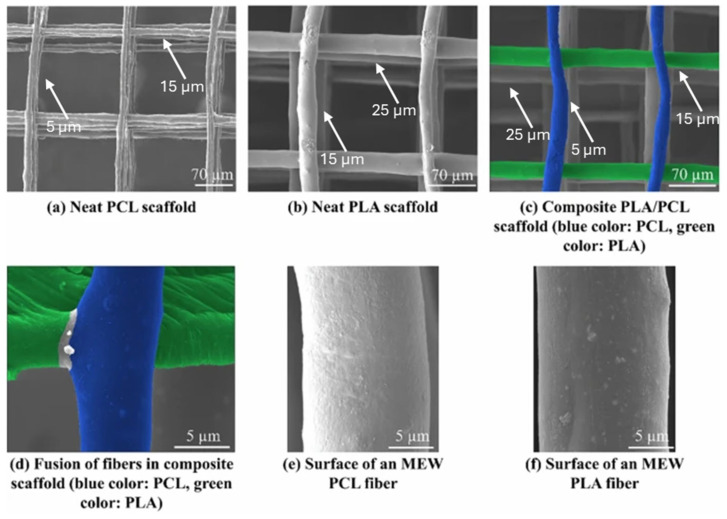
(**a**–**c**) Scanning electron microscope demonstration of bare PCL, bare PLA, and composite scaffolds. (**d**) The demonstration of bonding and connection of two fibers, where PLA is below and PCL is above. (**e**,**f**) The meltelectrowritten fiber is in two consecutive layers, which are perpendicular to each other with their specific surfaces. The performance of the material increases when they are in composite form compared to pristine form.

The collector strategy is not merely a geometric convenience. Rather, it directly administrates electric-field distribution and deposition stability. Prior work emphasizes that non-planar collectors or molds, as well as sacrificial/support materials, can enable non-planar fiber deposition, albeit with continuing challenges in maintaining high placement precision over complex topologies. MEW [154] enables concurrent tuning of fiber diameter and pore dimensions via coupled control of process parameters and toolpath. For instance, MEW scaffolds have been produced with nearly uniform pore sizes in the ~140-150 µm range using PLA/PCL systems, while also demonstrating that PCL and PLA can produce markedly different microfiber diameters, reflecting different melt viscosities and charge behavior [155]. Similarly, high-precision PLLA MEW scaffolds with defined filament and pore sizes have been systematically produced and evaluated for bone tissue engineering, illustrating that bio-based, thermally sensitive polymers can be processed when electrohydrodynamics and kinematics are carefully tuned [156]. Design freedom in MEW is always paired with a quality-control burden. Undesired fiber deviation modes, Taylor-cone fluctuations, and jet-lag-driven placement errors have been systematically categorized as key contributors to deteriorated printing accuracy, especially as architecture grows in complexity and layer count. Electrostatic interactions also scale with the number of printed layers as layer count increases, inter-fiber spacing constraints can emerge, and collector temperature has been shown to influence fiber disorder modes [17]. For example, Shahverdi et al. conducted a study on MEW by utilizing neat PLA, PCL, and composite PLA/PCL scaffolds [155]. The researchers conducted optimization studies on printing parameters to obtain scaffolds with nearly equal pore sizes in the range of 140 µm to 150 µm, which were successful. However, the obtained PCL fibers demonstrated narrower characteristic diameters in the range of 5 µm to 15 µm, compared to PLA fibers, which were between 15 and 25 µm, as shown in Figure 9. The precise positioning of PLA fibers was seen as difficult, most probably because of the higher viscosity of PLA melt compared to PCL melt. On the other hand, the printed composite scaffold of both PCL and PLA demonstrated not only a well-ordered box structure but also improved mechanical properties.

### 6.3. Mechanical and Biological Performance of MEW Scaffolds

MEW scaffolds are particularly valuable for tissue engineering because architecture is a first-class design variable that can be used to prescribe mechanical anisotropy, compliance, and contact guidance cues. Ordered microfibers provide a structural template that can control cell orientation and neo-tissue organization, while fiber spacing and laydown angles can be tuned to match target tissue mechanics more closely than many stochastic fibrous constructs [157]. Santschi et al. studied acetabular labrum restoration, MEW PCL scaffolds with varying architectures exhibited Young’s moduli spanning approximately 0.157 ± 0.057 MPa, with tensile behavior modulated by architecture [158]. Importantly, wave-patterned constructs were able to reproduce nonlinear elastic behavior closer to native labrum tissue, highlighting MEW’s capacity for mechanical programming via geometry. Likewise, vascular-mechanics-mimicking MEW architectures have been demonstrated to recapitulate aspects of tunica media mechanics and to guide preferential neo-tissue orientation, an example of how scaffold geometry can be designed to emulate tissue-specific microstructural organization and functional mechanics [159]. MEW’s ordered micro-architecture can promote cell alignment and spatially controlled tissue formation. In the labrum scaffold study, primary labrum cells were reported to migrate into the scaffold and grow in vitro, and reductions in fiber diameter and interfiber spacing improved cell distribution and spreading, although the relationship between mechanical optimization and cell compatibility can be architecture-dependent under dynamic loading. For bone-oriented applications, MEW scaffolds printed from bio-based PLLA have been evaluated in vitro with KUSA-A1 cells, supporting the premise that MEW can deliver mechanically stable, geometrically defined porous environments suitable for osteogenic studies when process–structure relationships are engineered appropriately [160]. Although MEW is frequently implemented with PCL due to printability and processing accessibility, biological performance often requires additional consideration of surface chemistry, degradation kinetics, and composite strategies. The feasibility of printing PLA, PCL, and PLA/PCL composites using MEW has been demonstrated, with composite architectures reported to improve mechanical properties and cell–scaffold interactions compared to neat components, underscoring the role of material selection and blending as a lever for both performance and translation [155]. Table 2 shows the general map for the MEW technique in tissue engineering applications. Although MEW is often discussed in terms of microarchitectural precision and in vitro cell guidance, recent in vivo studies have begun to validate its translational relevance. For example, bioglass-laden PCL scaffolds produced by MEW remained biocompatible after implantation in rats and promoted angiogenesis relative to pristine PCL scaffolds. Nevertheless, recent reviews consistently note that most MEW implantation studies still rely on small-animal models, and that clinically persuasive evidence will require large-animal studies, longer follow-up, and defect-specific functional testing [161].

### 6.4. Integration of MEW with Other Green Techniques

A key translational theme is that MEW is increasingly used not as a stand-alone process, but as a precision microfiber module within hybrid, greener workflows that combine scalable shaping with micro-architectural control. Combining MEW with solvent-free material extrusion/FDM can unlock multi-scale architectures. FDM provides robust macro-geometry and higher throughput, while MEW provides microfiber-level guidance and controlled porosity. A representative MDPI study demonstrated, for the first time, tandem use of FDM-printed reusable molds and MEW deposition of PCL to fabricate branched, hollow microfibrous constructs for vascularization, followed by assembly into lumen-containing structures and successful fibroblast growth over extended culture [165].

Hydrogel-fiber composites are one of the most impactful integration routes because they preserve hydrogel biofunctionality while addressing mechanical weakness [166]. Größbacher et al. emphasize that MEW can be combined with hydrogels to tailor multi-material constructs, where embedded MEW frames can substantially increase construct stiffness/toughness while occupying only a small volume fraction, potentially enabling lower hydrogel cross-linking densities that better support cell motility and tissue formation [166]. Consistent with this rationale, MEW-hydrogel hybrid scaffolds have been explored for thick tissue substitutes. For example, MEW–gelatin hybrid constructs have been reported as promising for replicating mechanical and physicochemical requirements relevant to full-thickness skin substitutes [167]. Green integration can also mean reducing auxiliary materials and steps while expanding geometry. Sacrificial/support materials like hydrogels used to guide deposition on complex topographies have been used to enable printing on non-planar surfaces, pointing to a pathway where water-based or easily removable supports allow complex structures without solvent-heavy post-processing. Positioning for this review: In the context of green and scalable manufacturing, these hybrid routes should be discussed as process intensification strategies, leveraging solvent-free macro-shaping and MEW micro-precision to achieve hierarchical scaffolds with fewer wet-chemical steps and clearer quality-control pathways.

### 6.5. Challenges in Scaling up MEW

Despite its precision and solvent-free nature, MEW faces well-recognized barriers to industrial translation. Key challenges include low throughput, poor reproducibility across users and environments, and the difficulty of navigating a nonlinear, highly coupled parameter space without robust process models and controls. MEW is fundamentally a serial deposition process by producing clinically sized, high-volume scaffolds with micron-scale fibers can be time-intensive. Moreover, scaling to many layers introduces electrostatic and thermal-history complications, which may force compromises in fiber spacing or require sophisticated process compensation. Quality loss manifests through measurable modes such as fiber deviation, jet-lag errors, and Taylor-cone instability [168]. These issues are sufficiently prevalent that recent reviews explicitly center design and quality improvement around stabilizing Taylor-cone behavior, quantifying jet lag, and mitigating fiber placement errors through synergistic tuning of process parameters and collector strategies. A promising path toward industry readiness is automation and closed-loop control, where in-line monitoring of jet behavior and deposition outcomes can reduce user dependence and improve reproducibility. Recent work demonstrates AI-enabled monitoring and control of MEW via computer vision and machine learning, explicitly targeting throughput limitations and reproducibility by creating data-driven models and feedback controllers that reduce output error in real time [151]. Hardware innovations, such as multi-axis kinematics, can expand design freedom and potentially reduce manual handling and assembly by enabling direct printing onto anatomically relevant collectors. Parallelization (multi-nozzle) is an intuitive throughput solution at the broader EHD-printing level. However, it introduces electric-field crosstalk and jet–jet interference challenges that require careful electrostatic and kinematic engineering. Therefore, it is valuable to frame MEW scaling not as a single-step make-it-faster problem, but as an integrated strategy combining material/rheology design for stable jets, model-based parameter selection, in-line monitoring and closed-loop control, and hardware parallelization and/or multi-axis automation.

## 7. Comparative Evaluation of Green Manufacturing Strategies

Eco-friendly tissue engineering scaffold fabrication techniques have received more attention in recent years. A comparison of solvent-free processing, scCO_2_ processing, and MEW is shown in Table 3 to illustrate the benefits and drawbacks of representative green manufacturing techniques. Before comparing the manufacturing platforms discussed, it is vital to clarify that solvent-free, green, and scalable are not interchangeable evaluation axes. In this review, solvent-free means the lack of organic solvents during the main scaffold formation; green means reducing hazards, waste, process intensity, and lifecycle burdens; and scalable means that the manufacturing process can be repeated while producing a product that has consistent quality attributes at a larger volume for realistic economic purposes. As a result, a particular process may excel in one area but may not do so in others. Again, for example, melt electrowriting is completely solvent-free and produces an exceptionally accurate architectural structure or assembly, but it is limited by its throughput, while melt-based techniques tend to be among the most scalable techniques; however, the overall environmental impact of melt-based processes depends on temperature, energy demand, source of materials, and post-processing characteristics. CO_2_-based materials theoretically or at least practically limit the amount of solvent used and allow for processes with fewer residues; however, the overall green impact of CO_2_ processing depends on the equipment used, the pressures involved, and the integration of downstream processing.

Scaffold morphology, mechanical performance, scalability, and intended biomedical application are some of the aspects that influence the choice of a suitable green manufacturing technique. Table 3 summarizes that whereas scCO_2_ processing allows for highly porous structures appropriate for drug delivery applications, solvent-free processing offers a straightforward and scalable production technique. On the other hand, MEW is very appealing for advanced tissue engineering scaffolds because it provides better mechanical tunability and architectural precision.

## 8. Hybrid Manufacturing: Combining scCO_2_, MEW, and Melt Processing for Hierarchical Scaffolds

Hybrid manufacturing is increasingly emerging as a design necessity rather than a mere process preference, because no single green fabrication route can simultaneously deliver patient-specific macroscopic geometry, deterministic and interconnected macroporosity for tissue ingrowth and perfusion, ECM-relevant microscale fibrous cues, and additional micro-/sub-microporosity and functionalization without compromising either mechanics or manufacturability [169]. Additive manufacturing (AM) routes enable reproducible, digitally defined architectures. However, they are typically resolution-limited at the micro-porosity scale and often require supplementary strategies to create multi-scale transport pathways and bioinstructive surfaces. Within the green and scalable paradigm of this review, the triad of melt processing, including melt extrusion/FFF/FDM, melt electrowriting (MEW), and supercritical CO_2_ (scCO_2_), is particularly compelling. Melt processing offers throughput and structural robustness; MEW offers micron-scale, precisely positioned fibers for guidance and mechanobiological control, and scCO_2_ offers solvent-free foaming/impregnation with tunable physicochemical interactions. Crucially, the recent scCO_2_ literature explicitly emphasizes that scCO_2_ foaming alone is often insufficient to meet practical requirements [170]. Hence, there is growing emphasis on combined and hybridized processing routes to overcome pore-control and surface “skin layer” limitations.

### 8.1. Why Hybrid Scaffolds Are Needed

Active tissues are hierarchical across multiple length scales, and tissue regeneration typically requires a similar multi-level instruction set from the scaffold. For bone repair, for example, cancellous bones are characterized by high porosity with pore sizes commonly reported in the range of hundreds of micrometers, supporting fluid flow and tissue ingrowth [171]. Meanwhile, microscale and sub-microscale features modulate protein adsorption, cell anchorage, and osteoinduction. A key insight from the pore-architecture literature is that macroporosity and microporosity are not interchangeable. Macropores, often described as >100 µm, are widely associated with space-making for tissue ingrowth and vascularization, whereas microporosity, often <10 µm, contributes to increased specific surface area, permeability, and biologically relevant surface interactions [172].

From a manufacturing standpoint, this multi-scale requirement exposes the limitations of single-method green fabrication. FFF/FDM (melt extrusion AM) robustly delivers designed macropores and patient-specific shapes but has inherent resolution limits that make <10 µm pores difficult or impractical. MEW can precisely deposit micron-scale fibers, commonly reported around ~5–40 µm, with potential down to sub-micron levels under optimized conditions, enabling ECM-like microarchitecture and anisotropic guidance. However, MEW alone can be constrained by building size, deposition time, and the load-bearing capacity of purely microfibrous lattices. scCO_2_ foaming/processing can generate internal porosity and can tune polymer morphology via plasticization. On the other hand, there are challenges, including controlling pore size distribution, achieving robust interconnectivity, and avoiding a non-porous skin layer at the surface. Therefore, hybrid scaffolds aim to decouple competing requirements: macroscopic handling and mechanics can be provided by a melt-processed framework, microfibrous biological guidance by MEW, and additional micro-/mesoporosity plus solvent-free functionalization by scCO_2_.

### 8.2. MEW + scCO_2_ (Conceptual Integration Pathways)

Although scCO_2_ has already been widely leveraged for solvent-free foaming and impregnation, and MEW has matured as a precision micro-architecture platform, their direct integration remains an underexploited direction compared with more established scCO_2_ pairings such as particle leaching, freeze-drying, breath figures, and mold patterning. There must be robust pathways that align with green manufacturing and are defensible for industrial translation. The first one is scCO_2_ post-processing of MEW scaffolds for sterile-by-design functionalization. scCO_2_ is not only a foaming agent but also a swelling/plasticizing medium that can promote impregnation of polymers with bioactive compounds. Furthermore, scCO_2_ has been discussed as a sterilization-enabling technology under specific conditions [173]. A particularly translation-ready concept is to treat MEW scaffolds as precision templates and then apply scCO_2_ as a downstream unit operation to deliver one or more of the following: First, drug/growth factor loading by scCO_2_-assisted impregnation, exploiting polymer swelling and CO_2_ tunability [174]. One-pot sterilization and processing, where feasible; studies demonstrate integrated scCO_2_ sterilization/foaming protocols and, importantly, a one-step process yielding sterile, drug-loaded PCL scaffolds while supporting cell attachment and proliferation [175]. When MEW is paired with scCO_2_ as a post-processing step, dimensional stability becomes a primary constraint because scCO_2_ can plasticize polymers depending on conditions. Process windows should therefore be selected to preserve MEW fiber placement and pore geometry.

Second, scCO_2_ generation of secondary porosity in melt-processed struts, with MEW as a guidance insert. A powerful hybrid concept is to use melt processing like FFF/FDM or melt molding to create designed macroporous lattices, then apply scCO_2_ to introduce secondary micro-/mesoporosity within struts and finally use MEW to introduce aligned fibrous cues either within macrochannels or at interfaces where guidance is needed. This approach is strongly supported by the general principle that AM provides deterministic macro-architecture, while gas- or scCO_2_-based methods can deliver micro-porosity that AM cannot easily print [176]. A closely related proof-of-concept exists for AM and scCO_2_ integration. The multi-material FDM structures (PCL/PLA) have been combined with scCO_2_ and breath figures to achieve internal pores (~80–300 µm) and external pores (~2–12 µm) in PCL-containing regions, illustrating how scCO_2_-based processing can add a porosity tier to printed architectures [15]. While this example does not include MEW, it provides a direct blueprint for adding MEW as the third tier to produce a macro, micro, and microfibrous hierarchy.

Third, scCO_2_ as a solution to surface transport bottlenecks for hybrid constructs. A well-documented bottleneck in scCO_2_ foaming is the formation of a non-porous skin layer that can impede cell ingress and mass transport, motivating combined strategies. Breath figures (BF) are one example that can render this skin layer porous and improve through-thickness pore connectivity when used alongside scCO_2_ processing [177]. In hybrid constructs, this becomes relevant in two ways. First, if scCO_2_ is applied after MEW, skin-layer mitigation strategies or selective surface poration may be necessary to ensure the MEW-mediated guidance is not isolated from perfusion pathways. Second, if MEW is applied after scCO_2_ foaming, the surface roughness/porosity created (or removed) by skin-layer phenomena will affect MEW deposition stability and interfacial bonding.

### 8.3. Melt Extrusion/FFF + MEW

Hybridizing FFF/FDM with MEW is among the most practically demonstrated routes to hierarchical architectures, because both are melt-based and can be digitally integrated, while targeting complementary length scales. Recent work demonstrates a tandem strategy in which FDM-produced conductive PLA molds enable the fabrication of branched, hollow MEW scaffolds from PCL that would be difficult to realize by MEW alone [178]. The scaffold halves can subsequently be heat-melded to form hollow constructs and then mechanically/biologically tested. Beyond mold-based approaches, direct combination has also been reported for regenerative targets requiring spatially distinct microarchitectures. For osteotendinous junction regeneration, a PCL scaffold was produced using MEW and FDM to create aligned and orthogonal fiber regions, followed by region-specific modifications, including collagen coating and hydroxyapatite addition, to support lineage-specific differentiation [179]. Notably, Abdal-hay et al. explicitly frame FDM as providing a framework to compensate for the limited mechanical strength of MEW-only structures, highlighting a central rationale for hybridization.

The FFF/FDM layer at macro-scale defines the outer geometry, macro-pore network, fixation features, and handling robustness, which is critical for surgical workflows and load-bearing contexts. The MEW layer at micro-scale provides precisely positioned fibers that regulate pore geometry, anisotropy, and guidance cues. MEW’s ability to prevent random whipping and enable ordered deposition distinguishes it from solution electrospinning for architected scaffolds [19]. The FFF/MEW hybrid still faces nontrivial process–structure constraints, accurate registration between toolpaths, thermal management to prevent remelting or distortion of MEW fibers, and scalable approaches for 3D (non-planar) deposition or assembly. These are precisely the themes emphasized in MEW process optimization, where printing stability, jet lag/fiber deviation, and reproducible fiber placement are treated as core barriers to engineering-grade quality [180]. Importantly, the FFF/MEW hybrid becomes even more valuable when scCO_2_ is added as a third element, since FFF can define macroporosity, MEW can define microfibrous guidance, and scCO_2_ can introduce secondary porosity or enable solvent-free bioactive loading/sterilization as a downstream step, which can produce genuinely hierarchical and functionally integrated constructs.

### 8.4. Hybrid Workflow Design Rules

A recurring reason why hybrid scaffolds underperform is not the lack of promising unit operations. However, there is an absence of design-for-hybrid-manufacturing rules that anticipate process interactions like thermal history, CO_2_ plasticization, interfacial bonding, and pore hierarchy coupling. Therefore, there should be a practical rule set that can be explicitly articulated. First, the allocation of each process to a specific length-scale mission should be conducted. Macro-size (>100 µm to mm), geometry, fixation, macrochannels, and designed lattice porosity for melt extrusion/FFF/FDM can be utilized. Microfibrous qualities (≈5–40 µm fibers; ordered), guidance, anisotropy, and ECM-mimetic architectures to MEW can be utilized. Microporosity (<10 µm) and secondary porosity tiers, surface area/permeability tuning, and potential bioactive impregnation to scCO_2_ can be used. Secondly, the choosing process sequence to protect the most sensitive feature should be evaluated. A robust default sequence for green hybrid manufacturing is high-temperature steps first (melt extrusion/FFF/FDM, then MEW) and scCO_2_ later (foaming/impregnation/sterilization). This ordering minimizes the risk that later thermal operations remelt MEW fibers or erase scCO_2_-induced microstructures. Thirdly, the engineering around scCO_2_ plasticization and dimensional drift should be considered. scCO_2_ can reduce polymer Tg/Tm and melt viscosity via plasticization; while this is beneficial for foaming and impregnation, it can also distort fine architectures if not managed. Practical actions should include selecting exposure conditions that preserve fiber placement, using controlled depressurization rates, anticipating shrinkage/expansion in CAD compensation, and designing mechanical locks that constrain deformation during scCO_2_ treatment. Fourthly, treating interfaces as first-class design variables should be analyzed. Hybrid scaffolds are only as strong as their FFF/MEW and MEW/foam interfaces. Strategies reported in FDM-MEW include mold-assisted fabrication and heat-melding/thermal bonding to assemble complementary halves or layers.

For tri-technology hybrids, interface planning should include polymer compatibility, thermal windows for bonding without collapse, and targeted roughness/porosity for adhesion. Fifthly, matching the metrology with each hierarchical tier for quality-by-design. MEW quality metrics are fiber diameter uniformity, placement accuracy, defect taxonomy (e.g., deviation modes), and jet stability monitoring. scCO_2_ quality metrics are pore size distribution, skin-layer assessment, interconnectivity, and spatial selectivity in multi-material systems. Macro-architecture metrics are permeability/mechanics interplay, pore geometry adherence to CAD, and repeatability across batches, which are central design considerations in AM scaffold studies [181,182,183]. Sixthly, building the workflow around translation-friendly unit operations should be adjusted. Wherever possible, prioritizing steps that reduce solvents and handling, particularly those that compress multiple functions into one operation, is crucial.

From a translational standpoint, hybrid scaffold systems should ultimately be tested in anatomically and mechanically relevant defect models. Recent osteochondral studies using biomimetic 3D-printed biodegradable scaffolds in rabbits reported improved regeneration compared with traditional geometries and framed personalized morphology as a key translational advantage. At the same time, these studies also emphasized the need for larger defects, large-animal models, and longer-term safety/degradation assessment before clinical implementation [184].

### 8.5. Proposed Roadmap for Industrial-Scale Translation

To develop a green scaffold at the industrial scale, as demonstrated through this review’s comparative analysis, there should be a phased approach with defined stages leading to clinical applications, rather than directly scaling up laboratory protocols. The first stage is the application definition of the scaffold, which will define the tissues that will be treated, the ultimate clinical use (patient or commercial), whether the scaffold is patient-matched or manufactured at a standardized level, the degradation time frame for the scaffold, the mechanical targets for the scaffold, and sterilization strategies. It is imperative to define the application’s purpose, the class of material, and whether the scaffold will be used as an acellular implant or will evolve into a more complex bioactive or cellular-based construct, as the translational expectations for each vary by intended use and material class. In Stage 2, the platform and feedstock(s) will be selected. Medical-grade materials with traceable supply chains should be prioritized, and the manufacturing method utilized should meet the architectural mission of the scaffold. For example, melt processing is a solvent-free method that has high-throughput capabilities and allows the development of macro-architecture. Melt electrowriting (MEW) allows high-resolution (30 μm–100 μm) designs, while supercritical carbon dioxide (scCO2) processes can be utilized for secondary porosity development, impregnation of bioreactor scaffolds before patient implantation, and, where appropriate, for post-process sterility assurance and compatibility with the final packaging systems (sterile vs. non-sterile). For Stage 3 to transpire, processes for producing the scaffold must be defined under a quality by design (QbD) framework, including: determining critical process parameters (CPPs) and critical quality attributes (CQAs) for each layer of the scaffold, defining acceptable process windows for product and/or process variability, and the introduction of in-line or at-line controls to maintain dimensional accuracy, porous structure, pore interconnectivity of scaffold materials and molecular weight retention in combination with bioactivity levels as each becomes critical to maintaining product viability over time. Stage 4 includes the completion of pilot-scale manufacturing and design transfer into a medical device quality (MDQ) system, where laboratory processes will be expanded, and associated documentation will be prepared, to use a workflow that includes: preparing the required software, qualifying the required equipment for quality control, tracing material controls and conducting qualification activities through parameter optimization, post-processing activities, sterilization preparation or preparation of the required technical files for packaging, labeling, and final inspection approval work. Stage 4 also includes validation of the production process of additive-manufactured scaffolds that have a level of acceptance by inspection alone, resulting in the need for process validation activities and documented (where required) process-monitoring activities to ensure that each build, batch, and machine produces consistent scaffolds. Stage 5 includes generating all necessary evidence from bench and ex vivo testing, to defect-related small animal models, to long-term preclinical studies in large-animal models, and formal clinical investigations where applicable. Finally, Stage 6 involves defining a strategy for post-market lifecycle maintenance, including post-market surveillance and benefit–risk assessment, as ongoing and continuous activities rather than as an obligation at the end of the lifecycle. Overall, the most reasonable near-term industrial path for producing green scaffolds will likely be a hybrid platform, where high-throughput evidence-based melt processing methods for producing the bulk of the scaffold, MEW methods for the addition of structural features to scaffolds, and scCO_2_ processes are combined for the development of solvent-free secondary porosity, loading, and/or sterilization, resulting in a robust, manufacturable and clinically validated translation platform for green scaffolds.

## 9. Integration of Artificial Intelligence in Manufacturing

Artificial intelligence (AI) serves as a fundamental integrative tool to facilitate the manufacture of biodegradable scaffolds for industrial use by integrating material selection with scaffold design, process optimization, and quality control into a unified data-driven system. AI approaches support the development of reverse designs inintended to reduce experimental trial-and-error approaches by linking formulated scaffold characteristics to desired biological and mechanical scaffolds, including the mechanical performance of the scaffold.

Machine learning and deep learning have been utilized in the areas of tissue engineering and biomaterial development for optimizing the design of scaffold structures, predicting the mechanical performance of scaffold materials, and assisting in designing scaffolds with specific biomechanical and mechanobiological targets. In essence, AI serves as a practical means to facilitate the transition of the design of green manufacture of scaffolds from a descriptive model to that of a predictive and designed manufacturing model [185].

AI plays a significant role in the manufacture of highly coupled and nonlinear parameter spaces throughout the manufacturing process. The use of digital twins and machine-learning algorithms in additive manufacturing enhances the processes by providing improved thermal history simulations of build materials, as well as the evolution of material microstructures during the printing processes. The AI-enhanced methods can also detect defects in 3D-printed scaffolds after completion, assess the quality of parts following completion, select adaptive settings based on predetermined parameters, and provide predictive maintenance of automated manufacturing systems. In the case of melt electrowriting processes, the application of computer vision technologies, neural network models, and closed-loop feedback control systems enabled enhanced monitoring of the printing process by providing continuous real-time imaging of the jets produced, thereby improving the quality, reproducibility, and throughput of melt-electrowritten scaffolds. Similar AI-assisted techniques would apply to solvent-free extrusion and molding processes, as the relationships among temperature, rheology, cooling history, and structural fidelity of scaffolds are highly interdependent and complex [186]. The use of these same AI-based methods will be beneficial in future supercritical carbon dioxide manufacturing processes, as these manufacturing processes are extremely sensitive to the various histories of pressure, temperature, time, and depressurization.

Thus, it is imperative to view AI not as an independent addition to an existing manufacturing operation, but rather as a process enabler that can help to establish process windows; develop in-line monitoring systems; detect variations; and ensure consistency across batches throughout the manufacturing of scaffolds. While the use of AI in scaffold manufacturing is anticipated to be a promising opportunity, there are several conditions that must be met before the systematic application of AI to scaffold manufacturing becomes feasible. Standardized databases, documented information regarding the composition and processing of scaffold material, validated sensor networks and databases, confirmed interpretation of the machine-learning models, and a thorough experimental verification process are all prerequisites for implementing AI into the process of scaffold manufacture [187]. Parse data, low rates of transferability across various production platforms, and lack of access to long-term biological and animal datasets have limited the application of robotically manufactured scaffolds and their generalizability. Therefore, the most realistic application of AI in scaffold manufacture is augmentation rather than total replacement of current quality-by-design and risk-management methodologies by producing quicker and better virtual screening and smart, optimized processing and closed-loop control strategies in the manufacture of scaffolds. Future developments in AI-based scaffolding technologies will likely depend upon the combination of AI and physics-informed approaches to product development and automated experimental processes, coupled with the need for regulatory-focused validation strategies combined with manufacturing efficiency and translational credibility [188,189].

## 10. Future Perspectives and Research Priorities

Methods like solvent-free, supercritical CO_2_, and melt electrowriting, which were created to address the drawbacks of tissue scaffolds made using conventional manufacturing techniques, provide notable benefits in terms of sustainability and exact structural control. Even though there has been a lot of progress in this area, there are still several important issues that need to be resolved before they can be extensively used at the clinical and industrial levels. For instance, techniques like solvent-free and MEW were created at the laboratory scale and are challenging to implement on an industrial scale. Therefore, more studies need to be done on the scalability, process standardization, and reproducible production issues of green synthesis production methods. For instance, in terms of scalability, multi-scale modeling studies should be carried out more often, and the relationship between production parameters and the morphological, mechanical, and biological features of the tissue scaffold should be better understood. For any green production technique, a process–structure–biological response matrix should be established, and data-driven strategies should be designed.

The application of the scCO_2_ approach is another crucial element in the development of scalability. ScCO_2_ offers great potential for biological applications because of its low-temperature processing capability and lack of solvent residue. Increased process control and scalability are the biggest benefits this approach will provide in the future. In this situation, a high degree of control over scaffold morphology is made possible by careful modification of pressure, temperature, and decompression rate; pore size, distribution, and internal structure may be tuned consistently.

The loading and controlled release studies of bioactive compounds presents another difficulty in green synthesis production processes. For instance, it is challenging to keep an eye on the medications’ stability, homogenous distribution, and regulated release kinetics when utilizing solvent-free procedures since bioactive compounds are injected into the polymeric matrix without the use of any appropriate solvent. Approaches like multi-phase scaffold systems or functionalization following loading with supercritical CO_2_ should be further explored in order to address these difficulties, as stated in the review.

In the future, developing multifunctional tissue scaffolds requires the hybrid use of green manufacturing methods in addition to the development of new highly functional polymers. Existing biodegradable (natural and synthetic) polymers used in tissue scaffold production have many advantages and disadvantages. For example, although natural polymers exhibit high biocompatibility, they have poor mechanical properties, rapid degradation, and limited functionalizability. Synthetic biodegradable polymers, on the other hand, are biologically inert, produce toxic by-products when degraded, and have limited ability to mimic natural ECM. However, to overcome these disadvantages, it is necessary to develop hybrid and composite systems by leveraging the biocompatibility of natural polymers and the mechanical strength of synthetic polymers and combining them with advanced manufacturing techniques to develop smart and multifunctional scaffolds. At this point, moving beyond the use of PCL as the gold standard in precision manufacturing methods like MEW is a necessity. Advances in materials science will enable the processing of new polymers at MEW, paving the way for multifunctional systems that simultaneously optimize both the mechanical stability and cellular interaction capacity of tissue scaffolds [21,152].

Green and scalable production methods offer numerous advantages to tissue engineering applications. If truly “green synthesis” is the goal, sustainability metrics such as lifecycle analysis and energy consumption must also be considered. The success of all these processes will only be possible through interdisciplinary collaborations and holistic approaches to clinical needs.

## 11. Conclusions

Tissue scaffolds are one of the main components of tissue engineering and regenerative medicine applications. The methods utilized in the production of the scaffolds involve a wide range of areas, yet they suffer from environmental concerns. Therefore, there is a need for the emergence of methods that remove or minimize environmental damage and avoid toxic solvents in scalable production processes. In this case, green synthesis methods offer promising solutions for enhancing environmental sustainability without sacrificing biocompatibility. This study aimed to provide a comprehensive and detailed overview of green and scalable production techniques for biodegradable polymeric tissue scaffolds by explaining solvent-free processing, scCO_2_, and MEW techniques. The advantages and disadvantages of each method are discussed comparatively and supported by current studies. These methods can be considered the main candidates for minimizing environmental concerns and eliminating toxic solvent residues. In addition, they can provide more precise control over the architecture, mechanical performance, and bifunctionality of tissue scaffolds compared to current methods. The three methods of manufacturing described in this review suggest that current industrial implementation with solvent-free melt processing is most efficient. Solvent-free melt methods incorporate established technologies (extrusion and molding), allowing for both continuous and high-throughput production, scalable process controls, and already established quality-assurance protocols. Conversely, supercritical CO_2_ technologies are viewed as an attractive add-on manufacturing technology for many end-use applications (including porosity creation, impregnation, and terminal sterilization), although the wider usage of these technologies is restricted by the necessity of high-pressure equipment, the associated capital/utility costs, compatibility issues with polymers and CO_2_, and the predominance of batch-type processes in this technology space. Melt electrowriting, while providing exceptional precision in the creation of cell-guidance architectures, remains the least mature stand-alone high-volume manufacturing option at this time. Factors such as low throughput, material incompatibilities, environmental sensitivity, and reproducibility issues are major obstacles to the widespread deployment of this technology in the near term. As such, the most likely scenario for industrial use in the short term will be characterized by a hierarchical relationship between each of these techniques. Solvent-free melt processing will serve as the primary backbone of each process, while the addition of scCO_2_ and MEW processes will add secondary value when enhanced porosity, bioactive loading, sterilization, or microscale guidance are required. On the other hand, even though the superior features of the techniques and advantages exist, scaling up production, ensuring process reproducibility, and meeting clinical and regulatory requirements remain challenges. Future research had better focus on optimizing multifunctional scaffold designs by hybrid use of green and scalable manufacturing methods to increase industrial scalability and integrating sustainable manufacturing approaches. Extensively, the use of these modern green manufacturing techniques will enable the production of biodegradable polymer scaffolds efficiently, at an environmentally friendly and clinically implementable level. Thus, this concept still promises future clinical and industrial applications in tissue engineering and regenerative medicine.

## Figures and Tables

**Table 1:** Solvent-free techniques: performance, scale-up levers, and key constraints (with key CPPs and CQAs) [82,110,111,112,113].

Solvent-Free Route	Typical Achievable Architecture/Resolution	Scale & Throughput (Relative)	Strengths (Performance + Translation)	Key Constraints/Failure Modes	Key CPPs (Control Levers)	Key CQAs (to Verify Release + Performance)
Melt molding (compression/injection/extrusion) + porogen leaching (aqueous)	Open-cell porosity achievable; pore size mainly set by porogen size/shape & fraction. Compression-molding polymer/porogen composites can improve thickness control before leaching.	High (industrial polymer processing). Main bottleneck: post-leaching + drying cycle time.	Scalable, reproducible, cost-effective, compatible with established molding infrastructure. Porosity/pore size tunable via porogen design.	Thermal degradation risk (hot spots, long residence time). Not truly a single step due to leaching/drying. Risk of incomplete porogen removal and pore non-uniformity.	Molding temperature (relative to Tg/Tm), pressure, residence time; mixing/porogen dispersion; porogen size/fraction; leaching time/agitation; drying conditions.	Mw retention/crystallinity; residual porogen/moisture; pore size distribution + interconnectivity; mechanical properties; dimensional accuracy.
Material extrusion AM (FDM/FFF)(filament-fed or pellet/screw-fed)	CAD-designed, fully interconnected macropore networks. Example (PCL): channel size 160–700 µm; porosity 48–77%.	Medium (serial build). Scale via multi-nozzle/parallel printing; pellet/screw feeding reduces filament-supply bottleneck.	High architectural control + reproducibility; patient-specific constructs (image-to-CAD). No solvent removal step.	High processing temperature limits the co-printing of cells/thermostable factors. Anisotropy and nozzle-limited resolution; filament availability constraints (filament-fed).	Nozzle temperature; extrusion rate; layer height; raster spacing/angle; print speed; build orientation; cooling.	Strand diameter; pore size/interconnectivity; dimensional fidelity; mechanical anisotropy; thermal degradation indicators (Mw).
Solid-state/dense-gas foaming (CO_2_/N_2_) ± salt leaching/extractable porogen	Pore size: few µm → several hundred µm; porosity ~10% → >90% (parameter-dependent). Often closed-cell/skin layer unless combined with porogens; hierarchical pores possible (e.g., ~312 µm macro + ~38 µm micro).	Medium (batch pressure-vessel cycles). Scale is limited by vessel volume, cycle time, and gas management.	Avoids organic solvents and chemical blowing agents; tunable pore size/porosity via PT–t and depressurization rate.	Closed pores/poor interconnectivity + skin layer formation; architecture gradients from gas penetration. Less deterministic global architecture vs. AM unless combined with molding/AM.	Saturation pressure/temperature/time; depressurization rate; foaming temperature; porogen content/size; post-annealing.	Open porosity + permeability/interconnectivity; skin layer thickness; pore size distribution; mechanical integrity.
Microsphere-based scaffolds: thermal sintering (heat)	Bottom-up assembly; interconnected pores; compatible with pore-size gradients. Over-sintering can cause pore occlusion and loss of interconnectivity.	Medium (batch). Scale depends on microsphere production, packing uniformity, and thermal uniformity.	Simple, low-cost consolidation; shape-specific constructs. Microspheres support spatial modulation and can act as 3D culture substrates; inorganic fillers are feasible.	Thermal exposure can compromise heat-labile bioactives. Shrinkage/warpage and pore occlusion if sintering is excessive.	Sintering temperature/time (relative to Tg/Tm); heating/cooling rate; microsphere size distribution; packing density.	Degree of fusion; interconnected porosity; mechanical properties; dimensional stability; bioactive activity retention (if incorporated/post-loaded).
Low-temperature CO_2_ sintering (sub-critical/pressurized CO_2_ plasticization)	Surface-limited plasticization enables fusion below Tg/Tm while preserving particle shape. Potential for single-step consolidation; cell-seeding reported under specific conditions.	Emerging → Medium. Requires pressure equipment; scale limited by cycle time and CO_2_ handling/validation.	Avoids high temperatures and organic solvents; maintains shape-specific scaffolds. CO_2_ can act as processing aid (and may assist sterilization under some conditions).	Parameter sensitivity; limited process window. CO_2_ sterilization effect can constrain simultaneous cell loading at high pressures/long exposures.	CO_2_ pressure; exposure time; temperature; depressurization profile; particle packing.	Interconnected porosity; shrinkage; mechanical properties. Cell viability (if applicable) and residual processing effects.
Powder-bed fusion: Selective laser sintering (SLS)	CAD-defined, layer-by-layer; sub-mm feature capability. Properties depend on delivered energy density (laser power, scan spacing, beam speed). Example (PCL): 2 mm square channels, 700 µm struts; porosity 44.8–76.5%.	Medium–High (industrial AM exists). Scale via build volume/nesting; needs powder qualification and powder-removal steps.	Single-step fabrication of complex architectures; strong parameter control over microstructure. Not limited to filament feedstock.	Powder availability/consistency (medical grade); thermal degradation/warpage; trapped powder removal. Surface roughness and variability with energy density.	Laser power; scan speed; hatch spacing; layer thickness; bed temperature; powder PSD/flowability; atmosphere control.	Dimensional accuracy; porosity/interconnectivity; mechanical properties; Mw/crystallinity; residual powder/contamination.

**Table 2:** MEW literature map in polymer parameters and fiber applications.

Polymer System	Key MEW Parameters (Reported)	Fiber Outcome & Architecture	Porosity	Degradation Rate	Application/Validation (Headline Finding)	Ref.
PCL (poly(ε-caprolactone))	T = 85 °C; V = 4 kV; gap = 1.2 mm; nozzle = 25 G–30 G; P = 0.5–4 bar; collector velocity = 1.2 × CTS–15 × CTS (e.g., 484–1763 mm min^−1^ for 25 G).	Fiber dia ≈0.10–33.6 µm (tuned by nozzle/pressure/speed). Example constructs: 150 µm pores; controllable surface topography + crystallinity.	~70–85%	12–24+ months (Slow)	Parameter–structure map for tuning PCL micro-topography/crystallinity (design lever for mechanics/degradation/cell cues).	[162]
PCL (Mn ≈ 45 k) + 10 wt% HA–PCL (deep layer); cytokine-loaded PLGA microspheres (inkjet interlayers)	Barrel T = 65 °C; needle = 21G; P = 0.1 MPa (~1 bar); gap = 2–4 mm; V = 3 kV at 2 mm, +1.5 kV per +1 mm (≈3–6 kV); 10 layers; pores: 100 µm (surface), 200 µm (mid/deep).	MEW fiber scale reported as 1–20 µm; smooth fibers; zonal pore design (100/200 µm) + multi-layer composition.	~75–90% (Gradient structure)	Biphasic (PLGA degrades in 1–2 months; PCL/HA network persists >12 months)	Cartilage defect repair (rabbit): zonal scaffold + growth factor delivery; BMSC adhesion/proliferation/differentiation (in vitro) and improved repair (in vivo).	[163]
PCL (neat)	Nozzle T = 100 °C; flow = 10 µL min^−1^; gap = 5 mm; collector T = 60 °C; CTS ≈ 60 mm s^−1^; print speed ≈ 65 mm s^−1^; V = 2–2.5 kV (1st layer) → 4.5–5 kV (10th layer).	Fiber dia 5–15 µm; 10-layer box scaffolds; pore size ≈ 140–160 µm.	~70–85%	12–24+ months (Slow)	Cytocompatible ordered microfibrous scaffolds (L929, HUVEC); demonstrates PCL MEW on modified commercial FDM platform.	[155]
PLA (neat)	Nozzle T = 230 °C; flow = 10 µL min^−1^; gap = 5 mm; collector T = 60 °C; CTS ≈ 60 mm s^−1^; print speed ≈ 65 mm s^−1^; V = 3.5–4 kV (1st layer) → 6–6.5 kV (10th layer).	Fiber dia 15–25 µm; 10-layer box scaffolds; pore size ≈ 140–160 µm.	~75–80%	6–12 months (Moderate)	Head-to-head comparison vs. PCL (mechanics/roughness/cell response); demonstrates high-T MEW of PLA with stable stacking.	[155]
PLGA ± acetyl-tributyl-citrate (ATEC) plasticizer (10–20 wt%)	Needle = 25G (ID 0.26 mm); gap = 3.5 mm; nozzle protrusion = 0.5 mm; V = 5.0 kV (head) and −0.5 kV (collector); ambient 20 °C, RH 40%; T_syringe/T_nozzle = 165/144 °C (PLGA), 157/136 °C (PLGA10), 150/129 °C (PLGA20).	PLGA fiber dia drifted ≈ 20.6 → 27.4 µm during first 3.5 h; PLGA10 yielded ≈ 14.1 ± 1.7 µm (first 3.5 h) and improved stability; demonstrates plasticizer-enabled MEW window.	~70–85%	1–6 months (Noted as faster-degrading systems)	Expands MEW material palette toward faster-degrading systems; quantifies thermal history/diameter drift (translation-critical for reproducibility).	[164]
Bisurea-based segmented copolymers: (AB)n and (ABAC)n with PDMS and/or PPO-PEG-PPO segments (amphiphilic, physically cross-linked)	T = 100 °C; V = 3 kV; gap = 2.2 mm; P = 0.5–1 bar; collector speed = 500–2800 mm min^−1^.	Fiber dia 7.6 ± 3.0 µm (2800 mm min^−1^, 1 bar) to 59.0 ± 12.5 µm (500 mm min^−1^, 1 bar); 500 µm fiber spacing; accurate stacking up to ~20 layers; smooth fibers + strong inter-layer bonding.	>80% (High, due to large spacing)	Highly tunable based on block ratios	Tunable hydrophilicity/hydrophobicity; cytotoxicity + cell adhesion assessed; candidate for soft-tissue interfaces and self-healing fiber fusion.	[37]
PEOT-PBT (mesh design)	Designed pore gap = 400 µm; T = 195 °C; nozzle dia = 250 µm; P = 5 kPa; speed = 60 mm s^−1^; gap = 2 mm; V = 2 kV; 8 layers (printed in nitrogen atmosphere).	Fiber dia 19.4 ± 2.9 µm; gap 378.1 ± 9.4 µm.	~80–90%	Months to years (Depends on PEOT:PBT ratio)	Elastic scaffolds for soft tissue regeneration: NIH-3T3 viability; ~98.9% pore bridging at day 28; reduced α-SMA expression over time.	[36]
PEOT-PBT (semi-random design)	Designed strand gap = 800 µm; T = 195 °C; nozzle dia = 350 µm; P = 4 kPa; speed = 20 mm s^−1^; gap = 2 mm; V = 3.5 kV; 8 layers (higher V used to induce semi-random deposition).	Fiber dia 19.9 ± 2.1 µm; gap 438.6 ± 80.6 µm; wavy/randomized fibers via jet instability.	~85–95%	Months to years (Depends on PEOT:PBT ratio)	Mechanically compliant ‘semi-random’ architecture; enables softer constructs vs. regular mesh while maintaining micro-fiber scale.	[36]
PCL (control mesh in PEOT-PBT study)	Designed pore gap = 400 µm; T = 100 °C; nozzle dia = 300 µm; P = 25 kPa; speed = 13 mm s^−1^; gap = 3 mm; V = 6.15 kV; 8 layers.	Fiber dia 18.3 ± 1.9 µm; gap 378.2 ± 8.2 µm.	~80–90%	12–24 months (Slow)	Benchmark PCL conditions used for direct mechanical/biological comparison to PEOT-PBT elastomer scaffolds.	[36]

**Table 3:** Comparison of solvent-free processing, scCO_2_ processing, and MEW strategies.

Criteria	Solvent-Free Processing	scCO_2_ Processing	MEW
Environmental impact assessment	High; lessens chemical waste and gets rid of hazardous chemicals	Extremely high; CO_2_ is safe for the environment, recyclable, and non-toxic	High; little chemical waste; solvent-free method
Chemical purity	High; the absence of organic solvents	Extremely high; no solvent remains are left by CO_2_	High; solvent-free thermal processing
Energy requirements	Moderate (because of the heating procedures)	High (due to high-pressure requirements)	Moderate (because of electrical field management and heating)
Process complexity	Quite simple	More complex	Moderate complexity
Morphological characteristics (pore size, porosity)	Pore size is controlled and limited	High porosity and a network of interconnected pores	Pore architecture is extremely accurate and controlled
Mechanical properties (elastic modulus, strength)	Moderate	Moderate	Controllable fiber orientation and high mechanical strength
Biodegradation kinetics	Material dependent	Increased degradation as a result of increased porosity	Adaptable degradation based on scaffold design
Biocompatibility	Good	Excellent	Excellent
Operational cost	Low	High	Moderate
Industrial feasibility	High production speed	Moderate; specific instruments required	Increasing but still limited
Application-oriented method selection	Ideal for scaffolds made of biodegradable polymers	Especially appropriate for drug delivery systems and highly porous scaffolds	Excellent for bone, cartilage, and vascular tissue engineering precise scaffolds

## Data Availability

No new data were created or analyzed in this study.
